# Fail-safe mechanism of *GCN4* translational control—uORF2 promotes reinitiation by analogous mechanism to uORF1 and thus secures its key role in *GCN4* expression

**DOI:** 10.1093/nar/gku204

**Published:** 2014-03-12

**Authors:** Stanislava Gunišová, Leoš Shivaya Valášek

**Affiliations:** Laboratory of Regulation of Gene Expression, Institute of Microbiology ASCR, Videnska 1083, 142 20 Prague, the Czech Republic

## Abstract

One of the extensively studied mechanisms of gene-specific translational regulation is reinitiation. It takes place on messenger RNAs (mRNAs) where main ORF is preceded by upstream ORF (uORF). Even though uORFs generally down-regulate main ORF expression, specific uORFs exist that allow high level of downstream ORF expression. The key is their ability to retain 40S subunits on mRNA upon termination of their translation to resume scanning for the next AUG. Here, we took advantage of the exemplary model system of reinitiation, the mRNA of yeast transcriptional activator GCN4 containing four short uORFs, and show that contrary to previous reports, not only the first but the first two of its uORFs allow efficient reinitiation. Strikingly, we demonstrate that they utilize a similar molecular mechanism relying on several *cis-*acting 5′ reinitiation-promoting elements, one of which they share, and the interaction with the a/TIF32 subunit of translation initiation factor eIF3. Since a similar mechanism operates also on *YAP1* uORF, our findings strongly suggest that basic principles of reinitiation are conserved. Furthermore, presence of two consecutive reinitiation-permissive uORFs followed by two reinitiation-non-permissive uORFs suggests that tightness of *GCN4* translational control is ensured by a fail-safe mechanism that effectively prevents or triggers *GCN4* expression under nutrient replete or deplete conditions, respectively.

## INTRODUCTION

Translational control mechanisms represent one of the critical aspects of the overall regulation of gene expression. They operate either on a general level by shutting down translation of most of messenger RNAs (mRNAs) or utilize sophisticated *trans*- and *cis*-acting features to control protein synthesis of individual mRNAs. One such gene-specific regulatory mechanism exploiting the presence of short upstream uORFs in mRNA leaders (i.e. 5′ untranslated regions—5′ UTRs) of various genes is called reinitiation (REI). It is characterized by the ability of some of these short uORFs to retain 40S ribosomal subunits on the same mRNA molecule even after they have been translated and the large 60S subunit has been recycled by the ribosome recycling factors (reviewed in (1,2)). Such post-termination 40S subunits are then able to resume scanning downstream and upon acquisition of the new ternary complex (TC), composed of Met-tRNA_i_^Met^ and eukaryotic initiation factor eIF2 in its GTP form, they are primed to recognize the next AUG codon (either of a main ORF or the following uORF) and reinitiate translation.

By definition, a short uORF is an open reading frame present in the 5′ leader of an mRNA proximal to the main (genic) reading frame. It is composed of a start codon and an in-frame termination codon separated by at least one additional sense codon. Several bioinformatics and genetic studies revealed presence of at least one uORF in ∼50% of mammalian, 20–30% of plant and in up to 20% of fungal mRNAs (3–6). The significance of these findings is often supported by high evolutionary conservation of the annotated uORFs. Moreover, the existence of multiple uORFs in a single mRNA leader is usually enriched in certain subgroups of mRNAs encoding growth factors, transcription factors and proto-oncogenes (3,7). The drawback of these robust analyses is that despite their relatively high incidence, the functional relevance of the vast majority of these uORFs for translational regulation of mRNAs that carry them has never been experimentally validated. Generally, the presence of a short uORF imposes a functional barrier for sufficient expression of a downstream main ORF resulting in very low protein levels of a protein that it encodes. Importantly, this repressive effect of uORFs can be alleviated under some conditions such as various types of stress, when the presence of uORF may promote an increased expression of certain specific mRNAs that are otherwise silent.

In fact, there are many ways how short uORFs may contribute to the overall regulation of expression of a particular gene, with REI being one of the most intriguing processes among them to study. In general, its efficiency depends on four main factors: (i) time required for uORF translation (which is determined by the relative length of uORF and the translation elongation rate), (ii) its flanking sequences, (iii) translation initiation factors (eIFs) involved in the primary initiation event, and (iv) its distance to the next open reading frame (regardless if main or again short) (8–10). It was also shown that successful REI will only occur when eIF3 and eIF4F complex remain associated with the 40S subunit after uORF translation termination (9–13). This implies that REI after short uORFs is more likely to be efficient because the critical eIFs have not been lost as yet. One of the key prerequisites for potent REI is the acquisition of the new TC by the post-termination 40S ribosome moving downstream from the uORF stop codon in order to enable scanning of triplets incoming into the P-site for the next AUG start codon. In this way, longer intercistronic distances are favored because shorter ones may not provide sufficient time for rebinding the TC, which results in bypassing of the start of a next ORF (reviewed in (14)). The classical mammalian representative of this type of regulation is the stress response transcription factor ATF4. The best studied example by far, however, is represented by its yeast functional homologue, transcriptional factor GCN4, in which the uORF-mediated translational control allows a fast response primarily to the nutritional stress (14).

The mechanism of *GCN4* translational control involves four short uORFs present in its 5′ mRNA leader and is very sensitive to the TC levels. According to the recent model (1,14), the first of the four uORFs is efficiently translated under both nutritional replete and deplete conditions and after its translation the post-termination 40S subunit remains attached to the mRNA and resumes scanning. In non-stressed cells, when the TC levels are high, nearly all of the rescanning ribosomes can rebind the TC before reaching one of the following three uORFs, neither of which has been until now believed to support efficient REI. As a result, ribosomes terminating on any of these three uORFs undergo a full recycling step, which prevents them from reaching and translating the main ORF. Under starvation conditions, the GCN2 kinase phosphorylates eIF2, which suspends formation of new TCs in the cytoplasm. Consequently, post-termination 40S ribosomes traveling from the uORF1 stop codon downstream will require more time to reacquire the TC to be able to recognize the next AUG start codon. This will allow ∼50% of them to bypass all three supposedly inhibitory uORFs and rebind the TC downstream of uORF4 but still upstream of the *GCN4* start codon. Thus, whereas the global protein synthesis is significantly down-regulated under nutrient deplete conditions, protein expression of *GCN4* is concurrently induced.

The logical implication of this description of the *GCN4* translational control mechanism is that the abilities of the four *GCN4* uORFs to allow resumption of scanning after their translation differ. While uORF1 is clearly REI-permissive (allows efficient REI), the following three uORFs were generally considered to be REI-non-permissive. In some of the original studies, however, uORF2 actually seemed to behave in a similar way as uORF1, as it was also able to partially overcome the inhibitory effects of subsequent two uORFs under starvation conditions (15–17). Nevertheless, its true REI potential was never further investigated. Moreover, Hinnebusch *et al.* demonstrated that the *GCN4* mRNA containing only uORF1 and uORF4 relatively effectively recapitulates the entire translational control of *GCN4* (15,18), as if uORFs 2 and 3 were not needed. Hence, perhaps owing to the experimental simplification, the most of the subsequent studies on regulation of *GCN4* expression were carried out with the uORF1–uORF4 minimalistic system and only a little attention, if any, was paid to uORFs 2 and 3. The latter two were thus somewhat automatically categorized as REI non-permissive, like uORF4, and in this way, they feature in most, if not all, available models.

An intriguing question of what makes uORF1 so efficient in promoting REI has puzzled this research field for many decades and in spite of that, it is still not fully answered. The critical determinants were localized to the 5′ and 3′ sequences flanking uORF1 many years ago (19,20), however, the molecular details of their action have begun emerging into the light only recently. As for the 3′ sequences, it was originally proposed that in order to function properly, the uORF1 3′ sequences have to be AU-rich (the AU-content is ∼60%) to prevent strong base-pairing interactions with the 40S subunit to allow prompt resumption of scanning—the uORF4 3′ sequences were on the other hand shown to be GC-rich (19). However, this view was recently challenged (2) as we noticed that with the exception of uORF4 (AU-content ∼40%), the 3′ sequences of the other two *GCN4* uORFs (2 and 3) have even higher AU-content (∼85 and ∼70%, respectively), yet they were not considered to be REI-permissive (12). Hence, the molecular mechanism by which the 3′ sequences immediately following the uORF1 stop codon contribute to REI is still quite a mystery.

**Figure 1.**
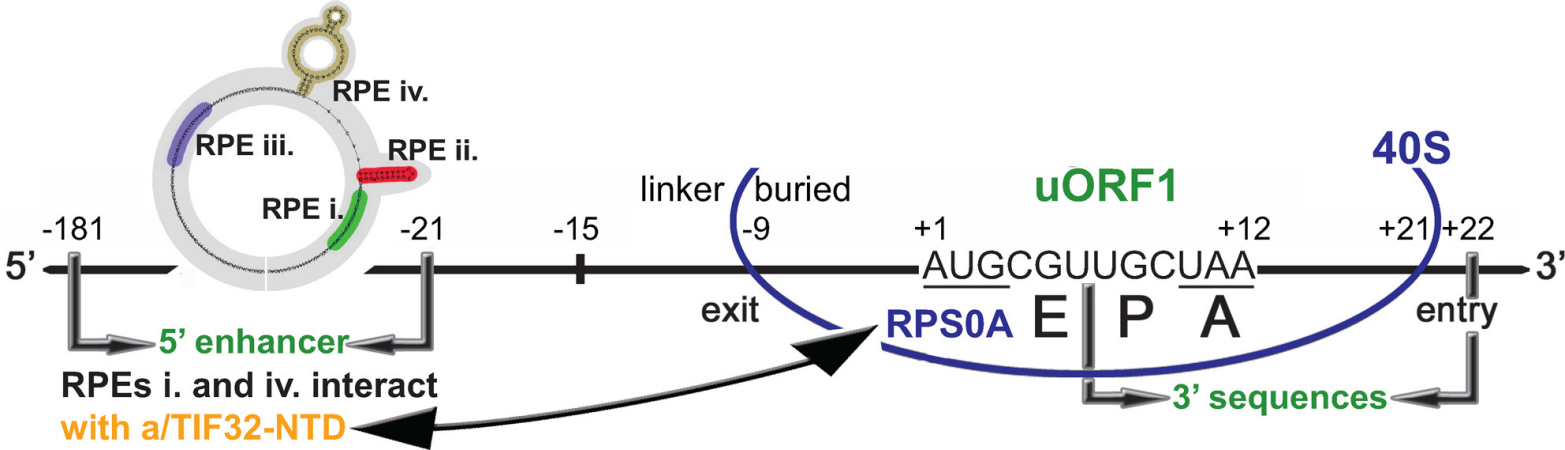
Schematic showing predicted position of the 40S ribosome terminating at the stop codon of uORF1 from the *GCN4* mRNA leader. The E, P and A sites of the 40S ribosome are aligned with the last two coding triplets and the UAA stop codon; entry and exit pores of the mRNA binding channel are labeled. The locations of the uORF1 5′ enhancer containing four RPEs, the RPEs i. and iv. of which interact with the NTD of a/TIF32, linker and buried parts of the sequences upstream of uORF1, as well as the 3′ sequences are indicated. The interaction between the a/TIF32-NTD and the small ribosomal protein RPS0A is depicted by a double headed arrow (adapted from (12)).

In contrast, the contribution of the uORF1 5′ sequences has been analyzed in great detail (10,12). First, a functional interaction between these sequences and the N-terminal domain (NTD) of the a/TIF32 subunit of the initiation factor eIF3 was identified and implicated in stabilizing the post-termination 40S subunits on the uORF1 stop codon to enable resumption of scanning for REI downstream (10). Later, four discernible REI-promoting elements (RPEs i.–iv.) were identified within this region, all of which together make up the so called 5′ enhancer (Figure 1) (12). Genetic epistatic experiments revealed that two of these RPEs, RPE i. and RPE iv., operate in synergy and in the a/TIF32-NTD dependent manner, whereas RPEs ii. and iii. contribute by a different, yet to be elucidated mechanism. Likewise, two separate regions within the a/TIF32-NTD were described and implicated in promoting REI in concert with RPEs i. and iv. (they were called Boxes 6 and 17 and each of them is composed of 10 amino acids (aa) residues). Taking into account the fact that the a/TIF32-NTD interacts with the small ribosomal protein RPS0A (21,22) occurring virtually at the 40S mRNA exit channel (23), it is highly likely, though not directly proven yet, that the a/TIF32-Boxes 6 and 17 directly interact with uORF1 RPEs i. and iv. Finally, a combination of computational and biochemical approaches revealed the 2D structure of the entire 5′ enhancer. The two key features of it are a 9-bp long bulged stem and a double-circle hairpin representing the RPEs ii. and iv., respectively (1). Collectively we proposed that these specific secondary structures have to fold progressively while the ribosome scans through them prior uORF1 translation in order to form fully active REI enhancer upon uORF1 termination. Strikingly, the structural motif similar to *GCN4* RPE iv. was also identified upstream of the REI-permissive uORF in the mRNA leader of yet another yeast transcriptional activator YAP1 (12). The fact that it likewise operated in the a/TIF32-NTD-dependent manner suggested that at least in yeasts the underlying mechanism of REI on short uORFs might be conserved.

To provide further evidence for the evolutionary conservation of the uORF1-like-mediated REI mechanism, we have wished to investigate more short uORFs with a potential to be REI-permissive. Intuitively, we started by revisiting the aforementioned initial studies, where *GCN4* uORF2 showed some REI promoting activity. Our rational was that if uORF2 proves itself to be truly REI-permissive, its proximity to uORF1 could imply that it also utilizes some of the aforementioned RPEs occurring upstream of its AUG start codon; in other words that it mechanistically does not differ from what we have described for *GCN4* uORF1 and *YAP1* uORF.

In this work, we clearly show that the solitary uORF2 is nearly as REI-permissive as uORF1. In addition, when combined with uORF4 in the minimalistic regulatory system (analogous to the one used routinely in the past with uORFs 1 and 4), it relatively effectively recapitulates the translational control mechanism of *GCN4.* Strikingly, the similarly high efficiency of REI promoted by uORF2 was found to stem from the same *modus operandi* shared by uORF1 and uORF2. In particular, we revealed that the REI competence of uORF2 strictly relies on: (i) the structured, eIF3-independent RPE ii. of uORF1, which thus represents a common REI-promoting element for both of these uORFs, and (ii) a specific, 10-bp long element designated as RPE v., which occurs in the vicinity of the 40S mRNA exit channel of the 80S ribosome terminating on uORF2 and, not surprisingly, operates in the a/TIF32-NTD-dependent manner. Thus, together with *GCN4* uORF1 and *YAP1* uORF, the *GCN4* uORF2 is the third short uORF that promotes REI by *cis*-acting elements upstream of its coding region, some of which functionally interact with the a/TIF32 subunit of eIF3. Our results therefore strongly support the idea of evolutionary conservation of general principles of translation reinitiation mediated by short uORFs at least among yeasts. Implications of the existence of two consecutive REI-permissive uORFs in the *GCN4* mRNA leader are discussed.

## MATERIALS AND METHODS

### Yeast strains, plasmids and other biochemical methods

Lists of strains (Supplementary Table S1), plasmids (Supplementary Table S2), and PCR primers (Supplementary Table S3) used in this study and details of their construction can be found in the Supplementary Data. β-galactosidase assays were conducted as described previously (19,24).

## RESULTS

### uORF2 from the *GCN4* mRNA leader resembles the well-established REI-permissive uORF1 in its high REI-promoting activity that is dependent on the N-terminal domain of the a/TIF32 subunit of eIF3

**Figure 2.**
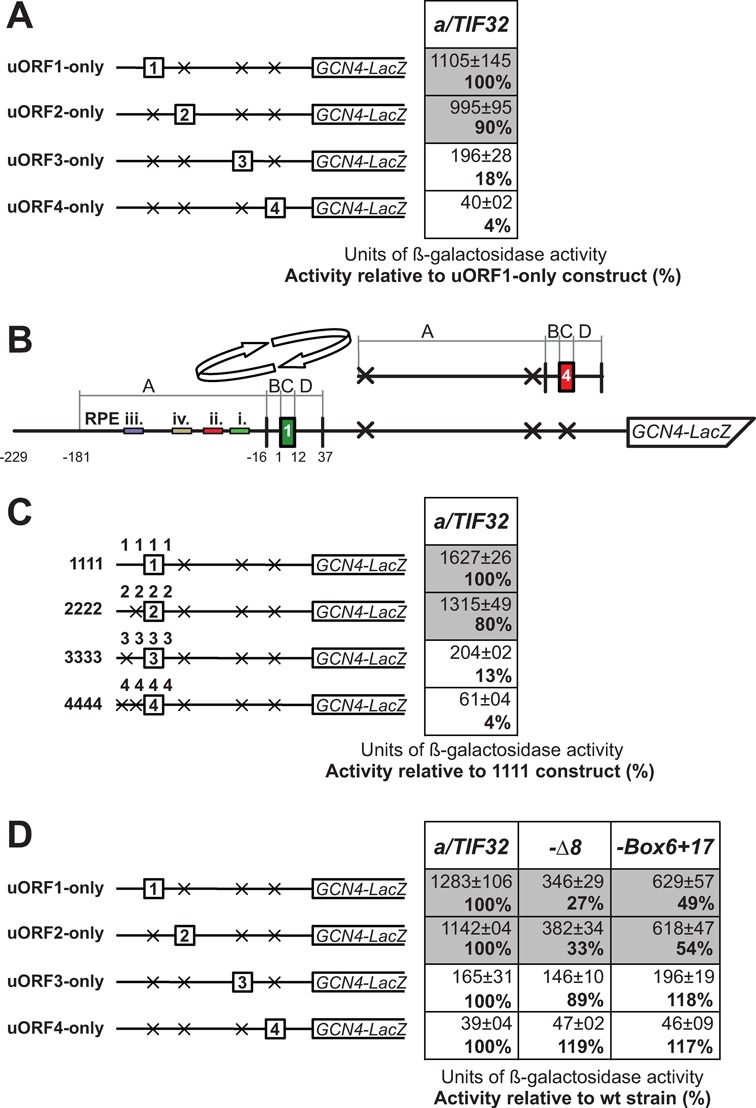
Solitary uORF2 promotes REI to the similar extent and by an analogous, eIF3-dependent mechanism as the REI-permissive uORF1. (**A**) Solitary uORFs from the *GCN4* mRNA leader in their native positions were introduced into the YSG2 strain. The resulting transformants were pre-cultured in minimal media overnight, diluted to OD_600_ ∼0.35, grown for additional 6 h and the β-galactosidase activities were measured in the WCEs and expressed in units of nmol of *o*-nitrophenyl-β-d-galactopyranoside hydrolyzed/min/mg of protein. The mean values and standard deviations obtained from at least three independent measurements with three independent transformants, and activities of the respective uORFx-only constructs relative to uORF1-only are given in right column. (**B**) Schematic showing the *GCN4-lacZ* construct containing solitary uORF1, the surrounding sequences of which were divided into four separate segments (A1–D1; see text for further details). Arrows indicate replacements of these segments with the corresponding segments (A4–D4) surrounding uORF4, shown to the right of the arrows. Colored bars indicate sequence positions of individual uORF1-specific RPEs. (**C**) Solitary uORFs 2–4 from the *GCN4* mRNA leader with their flanking sequences (in which start codons of other uORFs were mutated out; segments Ax–Dx) were placed in the native position of uORF1 and analyzed as described in panel (A). (**D**) The YSG2 (*a/TIF32*), YSG15 (*a/tif32-Δ8*), and YSG38 (*a/tif32-ΔBox6+17*) strains were introduced with the *GCN4-lacZ* constructs described in panel (A) and analyzed as described therein.

In order to examine the REI potential of the questionable *GCN4* uORF2 in a comprehensive manner, we first decided to compare REI activities of all four *GCN4* uORFs separately. To do that, we created two sets of *GCN4-lacZ* fusion constructs: one containing solitary uORFs in their natural positions in the *GCN4* leader (Figure 2A, referred to as ‘uORFx-only’); and the other replacing uORF1 and its flanking sequences (segments A, B, C and D—see the schematic in Figure 2B) with any of the three remaining uORFs and their corresponding flanking sequences (referred to as ‘xxxx’ in Figure 2C) according to (12). In detail, segment A is 166 bp in length (from position −181 to −16 relative to the uORF1 AUG start codon) and corresponds to the 5′ REI-promoting sequences of uORF1; segment B is 15-bp long (−15 to −1), designated previously as the linker that is buried in the terminating ribosome (10); segment C contains coding triplets and the stop codon; and segment D encompasses 25 bp immediately following the stop codon, which correspond to the putative 3′ REI promoting sequences of uORF1 (19). (Please note that the schematic in Figure 2B depicts these replacements for illustrative purposes only for uORF4.) In both sets of these constructs, the start codons of all other uORFs except the one under study were mutated out. The β-galactosidase activities of each set were measured as described previously (19) and the mean values (with standard deviations) were expressed relative to the values obtained with the REI-permissive uORF1 constructs that were set to 100%. Please note that throughout this study, all measurements were always performed in at least three independent experiments with minimally three (but usually five) individual transformants in triplicates (or pentaplicates) for each construct. It is also important to note that mRNAs produced from all *GCN4-lacZ* constructs used throughout the study are highly stable in both a/TIF32 wt and mutant strains thanks to the fact that they all contain an intact stabilizer element (STE) that protects the natural *GCN4* mRNA from the nonsense-mediated decay (NMD) pathway (10,12,25).

Consistent with our earlier study (12), both constructs carrying the solitary REI-non-permissive uORF4 displayed the identical 4% background REI activity when compared with the solitary uORF1 constructs (Figure 2A and C). Strikingly, whereas uORF3 allowed between 13 and 18% of the relative REI activity (i.e. ∼4-fold higher than uORF4), uORF2 reached unexpectedly high 80–90% of the relative β-galactosidase activities (Figure 2A and C). Importantly, the initiation rates on AUGs of both uORF1 and uORF2 were virtually the same (data not shown). Given the relative proximity of uORF2 to uORF1 (the distance between their AUGs is 68 nt), it is possible that uORF2 makes a use of the uORF1-specific RPEs and/or utilizes some yet to be identified RPEs on its own, which would be situated in the intercistronic region (see below). In any case, these findings strongly suggest that, in contrast to the textbook models, uORF2 is indeed a REI-permissive uORF with the similar REI capacity to well-characterized uORF1, whereas uORF3 is rather REI-non-permissive, as described in the literature, however, with a markedly higher REI potential than uORF4.

Next, we asked whether the full REI competence of uORF2 also depends on its functional interaction with the a/TIF32-NTD like in case of uORF1 (12). Hence, we carried out the analysis of the ‘uORFx-only’ set in strains bearing the REI-deficient *TIF32* alleles, either lacking the first 200 aa of their N-terminus (in *a/tif32-Δ8*) or harboring two blocks of 10 alanine substitutions (aa regions 51–60 and 161–170; in *a/tif32-Box6+17*), into which the interaction between uORF1 RPEs and a/TIF32-NTD was specifically mapped. To compare the changes in the behavior of individual uORFx-only constructs between wt and mutant a/TIF32 alleles, the values measured in a/TIF32 wt strains were set to 100% this time. In a striking analogy with the previously described uORF1 effects (12), the uORF2-only construct decreased β-galactosidase activities in both mutant strains by a similar number (∼70% in *a/tif32-Δ8* and ∼50% in *a/tif32-Box6+17*) (Figure 2D). In contrast, no significant changes were measured with the uORF3-only construct as well as with the negative control in uORF4-only. Together these findings strongly suggested that the REI-permissive uORF2 might mechanistically operate in a very similar fashion as uORF1.

### The RPE ii. of uORF1 serves as a common, eIF3-independent REI-promoting element for both uORF1 and uORF2

**Figure 3.**
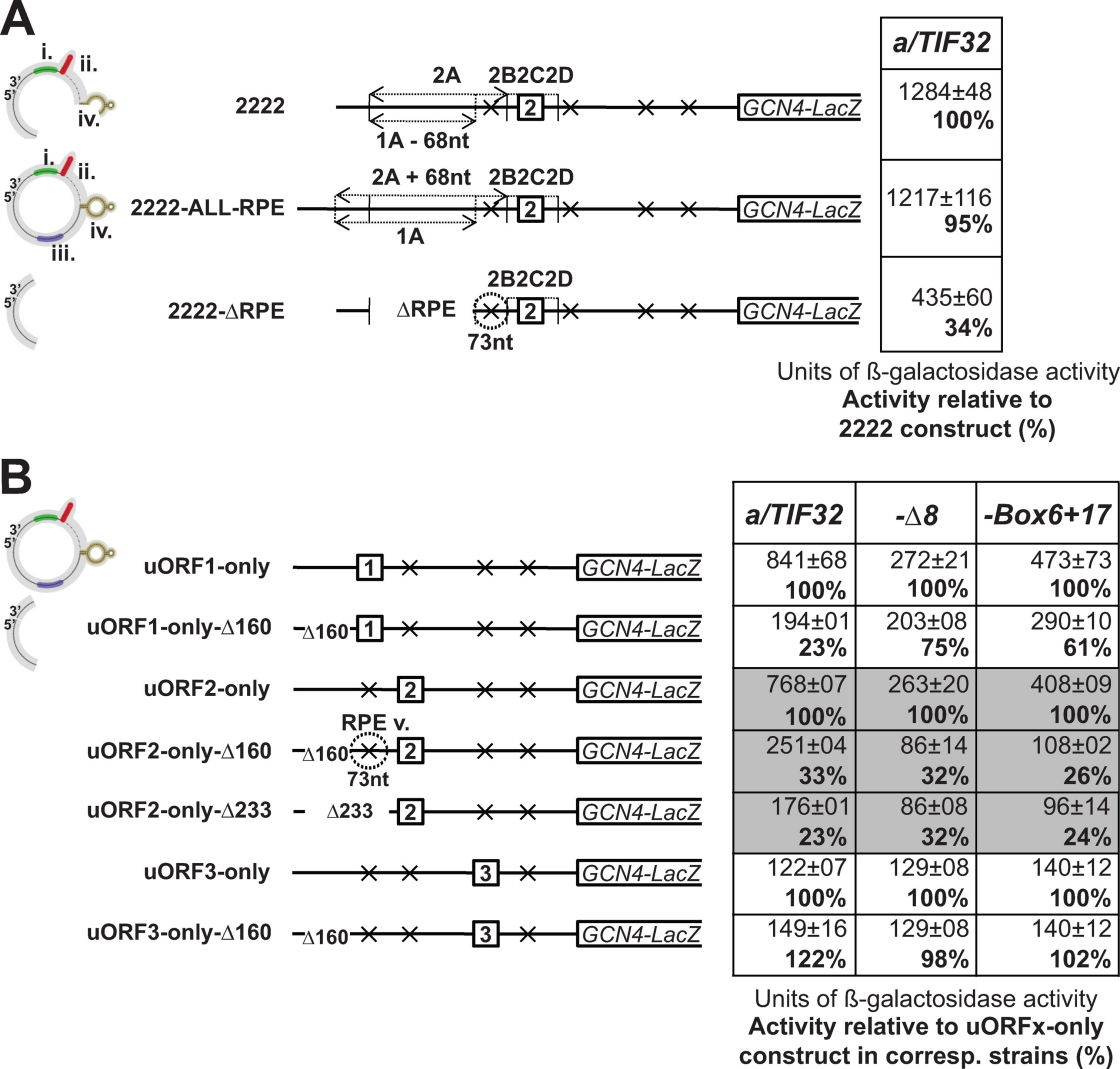
High REI potential of uORF2 depends on the downstream half of the uORF1 5′ enhancer and sequences immediately preceding uORF2 that show an epistatic genetic interaction with mutations in the a/TIF32-NTD. (**A**) The A segment of the 2222 construct from Figure 2C was modified as illustrated in the schematics to the left of the table and the resulting constructs were introduced into the YSG2 strain and analyzed as described in Figure 2A. The uORF1 5′ enhancer schematics at the very left of the figure illustrate the impact of these modifications on the presence or absence of uORF1-specific RPEs in individual constructs. (**B**) The indicated uORFx-only constructs were deleted for the uORF1-specific RPEs (Δ160), with the exception of uORF2-only construct that was in addition deleted also for the sequences immediately upstream of its linker region (additional 73 nt containing the uORF2-specific RPE v.) in the Δ233 construct, and the resulting constructs were introduced into YSG2, YSG15 and YSG38 strains and analyzed as described in Figure 2A.

The fact that uORF2 allows efficient REI only in the presence of the intact a/TIF32 protein, the NTD of which was shown to specifically interact with the uORF1 RPEs i. and iv., prompted us to examine whether the same elements might be involved in the REI promoted by uORF2. Since the 2222 construct lacks the entire RPE iii. and also a part of RPE iv., which were removed by the A segment swap between uORF2 and uORF1 (altogether 68 nt; Figure 3A, region 1A-68nt), we first recreated the full uORF1 5′ enhancer in the 2222 construct by adding back the 68 nt fragment containing intact RPEs iii. and iv. (Figure 3A, construct 2222-ALL-RPE). This modification had no effect on the uORF2 REI activity; however, suggesting that neither RPE iii. nor RPE iv. are involved (Figure 3A). In contrast, when we removed the 1A-68nt segment bearing intact RPEs i. and ii. from the 2222 construct, thus completely eliminating the entire uORF1 5′ enhancer (Figure 3A, construct 2222-ΔRPE), the REI activity decreased to ∼34%. This result strongly suggested that RPE i. and/or ii. might be indeed important for the uORF2 promoted REI.

As an independent proof of principle, we also removed the entire uORF1 5′ enhancer in the context of the uORF2-only construct by deleting the 160-bp long segment preceding uORF1, as described previously (10) (Figure 3B, construct uORF2-only-Δ160). As expected, the *Δ160* mutation caused a sharp drop in REI activity of the uORF1-only-Δ160 (to ∼23%) as well as uORF2-only-Δ160 (to ∼33%) constructs, the latter of which perfectly matched the values obtained with the 2222-ΔRPE construct (please compare panels A and B in Figure 3). On the other hand, no changes in β-galactosidase activities were seen when the *Δ160* mutation was introduced into the uORF3-only construct (Figure 3B).

We noticed that the effects caused by the *Δ160* mutation were repeatedly stronger (by ∼10%) in the uORF1-only versus uORF2-only constructs. Even though this difference may seem relatively insignificant, it has been observed in at least five independent measurements with iron discipline. This could indicate that the sequences immediately preceding the linker (segment B) of uORF2, which were not removed by the *Δ160* mutation, could also contribute to the uORF2 REI potential as its specific feature. To test this, we deleted the region preceding the uORF2 linker (comprising 73 nt) together with the *Δ160* region (Figure 3B, construct uORF2-only-Δ233), hence in principle removing the entire 2A+68 segment as shown in Figure 3A. Indeed, we observed that this progressive deletion evened the dramatic reductions in the REI activities between uORF1-only-Δ160 and uORF2-only-Δ233 constructs, as they both reached identical 23%—again with compelling regularity. Importantly, this ultimate drop in the uORF2 REI activity to the uORF1-only-Δ160 level was seen only in the presence of the wt a/TIF32 protein and not with the *a/tif32-Δ8* or *a/tif32-Box6+17* mutations (Figure 3B), indicating a genetic epistasis interaction between these a/TIF32-NTD mutations and the 73 nt region preceding the uORF2 linker. This further suggests that within this 73 nt region indeed exists the uORF2-specific, eIF3-dependent REI promoting feature, which we designated as RPE v. (see further below).

**Figure 4.**
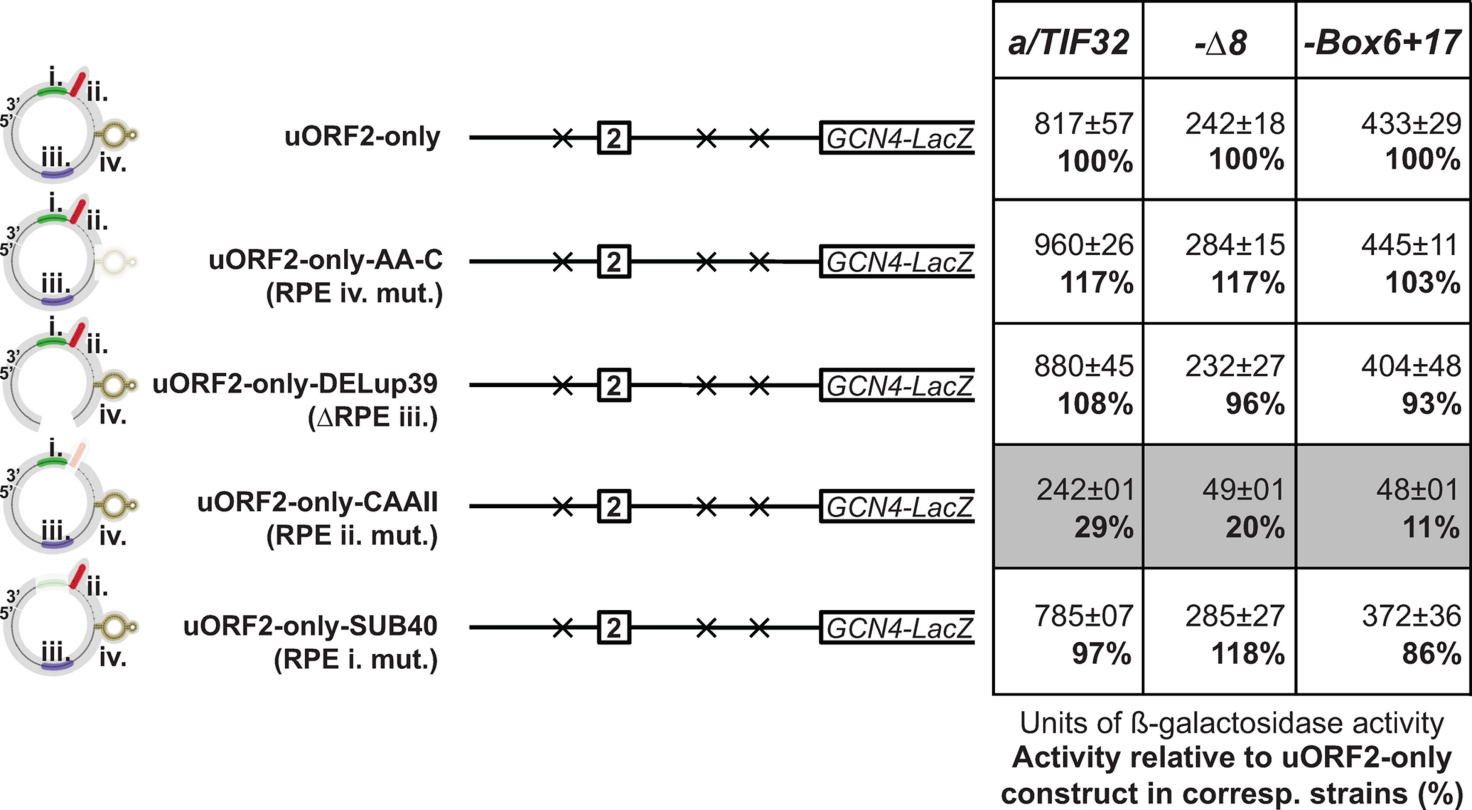
The RPE ii. of uORF1 serves as a common, eIF3-independent REI-promoting element for both uORF1 and uORF2. Previously identified mutations of the uORF1-specific RPEs (illustrated by the modified uORF1 5′ enhancer schematics at the very left of the figure) were individually introduced into the uORF2-only construct and the resulting constructs were introduced into YSG2, YSG15 and YSG38 strains and analyzed as described in Figure 2A.

As aforementioned, the robust reduction in REI activity caused by introduction of the *Δ160* and *ΔRPE* mutations into uORF2-only and 2222 constructs, respectively, strongly suggested that eIF3-dependent RPE i. and/or eIF3-independent RPE ii. of uORF1 might be directly involved in the molecular mechanism by which uORF2 promotes REI. To examine the contribution of the individual uORF1-specific RPEs in detail, we separately eliminated the REI-promoting effect of each of them by specific mutations that were generated and well characterized in our previous study (12). In particular, uORF2-only-AA-C disrupted the secondary structure of RPE iv., uORF2-only-DELup39 removed RPE iii., uORF2-only-CAAII nullified RPE ii. and uORF2-only-SUB40 altered the sequence of RPE i. Out of these mutations, only the CAAII mutation eliminating the RPE ii. led to a robust decrease in β-galactosidase activity (Figure 4). Consistently, the same specific effect was also seen when the CAAII mutation was introduced into the 2222 construct (Supplementary Figure S1). In contrast, no defects were observed for 2222-SUB40 (Supplementary Figure S1) and for AA-C and DELup39 mutations in the 2222-ALL-RPE construct (Supplementary Figure S2). Importantly, the observed robust decrease in REI activity by CAAII occurred even in the presence of both *a/tif32-Δ8* and *a/tif32-Box6+17* mutations clearly suggesting that it acts independently of the a/TIF32-NTD, as described before (12) (Figure 4 and Supplementary Figure S1). To conclude, the RPE ii. of uORF1 is the common *cis*-acting feature enhancing the REI potential of both uORF1 and uORF2 in an eIF3- and to some extent also distance-independent manner.

### In addition to RPE ii., the uORF2 REI permissiveness depends also on its own, eIF3-dependent RPE v. with shared sequence similarity to uORF1-specific RPE i.

**Figure 5.**
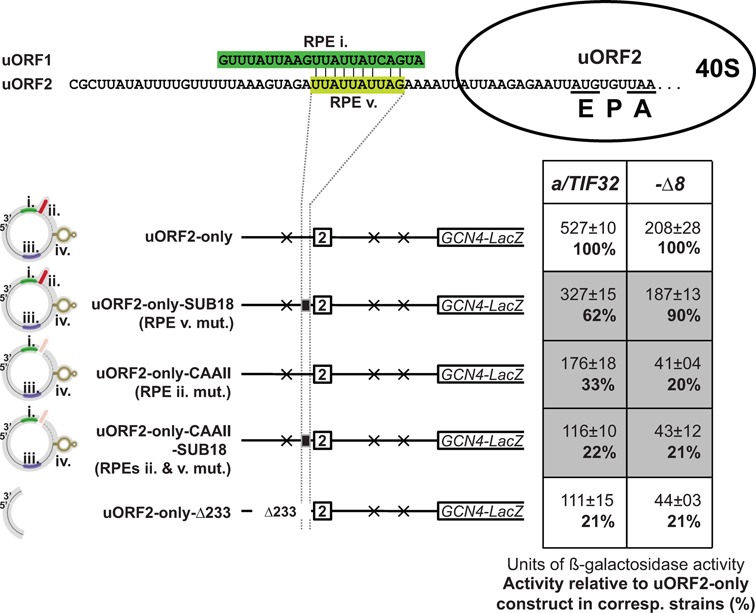
The uORF2 REI permissiveness depends also on its own, eIF3-dependent, sequence-specific RPE v., which shares sequence similarity to uORF1-specific RPE i. Sequence alignment of uORF1-specific RPE i. with the newly identified uORF2-specific RPE v. schematically depicted with the post-termination 40S ribosome with the uORF2 stop codon residing in its A-site. The previously identified substitution of the uORF1-specific RPE ii. (CAAII) and a newly generated substitution of RPE v. (SUB18) were individually or in combination introduced into the uORF2-only construct and the resulting constructs were introduced into YSG2 and YSG15 strains and analyzed as described in Figure 2A.

Next, we wished to specify the precise coordinates of the uORF2-specific RPE v. within the 73 nt long segment immediately preceding the uORF2 linker identified above (Figure 3B). The observed dependence of this feature on the intact NTD of a/TIF32 suggested that the action of RPE v. could be similar to the uORF1-specific, eIF3-dependent RPEs i. and/or iv. (12). Note that RPE i. is unstructured and sequence specific, whereas RPE iv. forms a stable double-circle hairpin (Figure 1). Since the *in silico* analysis of the 73 nt long segment containing RPE v. showed no predictions of any stable secondary structures, which is consistent with the fact that the whole inspected area is highly AU-rich (A 44%, C 5%, G 9% and T 37%), we turned our attention to the actual primary sequence. In particular, we used the 22-nt long sequence of the uORF1-specific RPE i. as a bait and identified one region encompassing 10 nt situated just upstream of the uORF2 linker, which was with the exception of one single nt identical to the second half of RPE i. (Figure 5). To test whether or not this short sequence motif constitutes the sought for RPE v., all of its nucleotides were mutated into complementary sequence in uORF2-only-SUB18. As shown in Figure 5, the SUB18 mutation did indeed reduce the β-galactosidase activity in the *a/TIF32* wt but not in the *a/tif32-Δ8* mutant strain. These findings thus define the important sequence determinants of RPE v. and, in addition, further corroborate its epistatic genetic interaction with the a/TIF32-NTD. Consistently, combining SUB18 with CAAII in the uORF2-only-CAAII-SUB18 construct produced an additive effect only in the *a/TIF32* wt strain, where the dramatically diminished β-galactosidase activity matched that of the complete deletion of all five RPEs in uORF2-only-Δ233 (Figure 5). In contrast, the REI activity in *a/tif32-Δ8* was at its lowest already with the uORF2-only-CAAII construct regardless the presence of the SUB18 mutation (Figure 5, compare uORF2-only-CAAII with uORF2-only-CAAII-SUB18). Together these results imply that the uORF2-specific RPE v. is (i) contained within the 10 nt region that has just emerged from the 40S mRNA exit channel when ribosome terminates on uORF2, (ii) operates strictly in the a/TIF32-NTD dependent manner (the NTD of a/TIF32 interacts with RPS0 occuring at the mRNA exit pore (22)), and (iii) together with RPE ii. represent the only two REI-promoting features that are responsible for the full REI capacity of uORF2.

### uORF2 operates in the minimalistic *GCN4* translational control system composed of only uORFs 2 and 4 in the similar fashion as uORF1

The fact that uORF2 is nearly as permissive for REI as uORF1 evokes an obvious question of what impact it may have on *GCN4* translational control in wt cells. To address this question we first decided to examine the *GCN4* inducibility by uORF2 in the minimalistic *GCN4* translational control system composed of only uORFs 2 and 4 under starvation conditions (AUGs of uORFs 1 and 3 were mutated out). The reason why we chose this system is self-evident; with uORF1 present in front of uORF2 it would be impossible to assess what contribution to the overall induction of *GCN4* expression uORF2 makes. uORF3 was left out because it was previously shown that such a minimalistic system composed of only uORFs 1 and 4 operates nearly as good as the intact *GCN4* leader (14).

**Figure 6.**
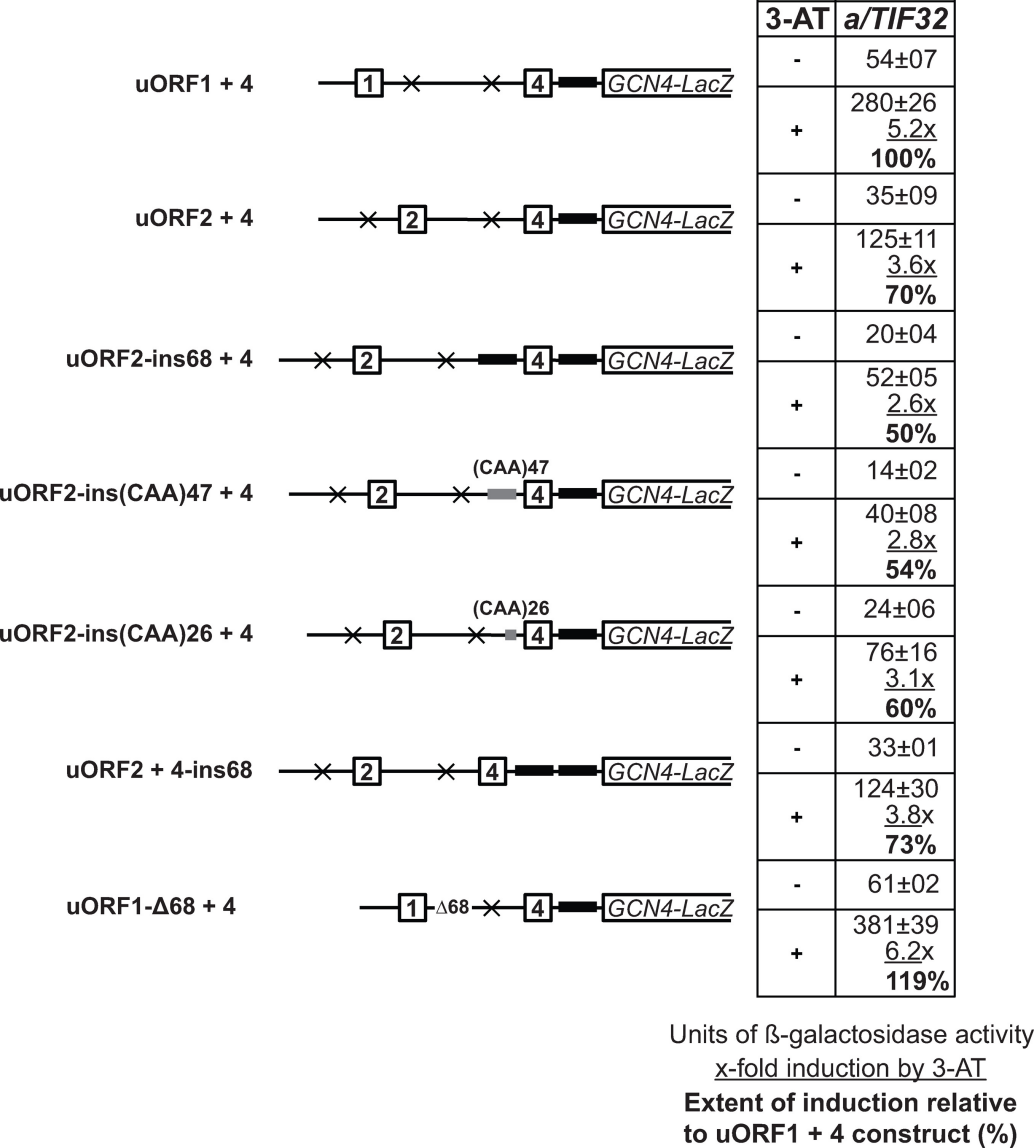
uORF2 operates in the minimalistic *GCN4* translational control system composed of only uORFs 2 and 4 in the similar fashion as uORF1. The construct carrying uORF2 and uORF4 was modified as depicted in the schematics to the left of the table. The resulting mutant constructs were introduced together with the construct carrying uORF1 and uORF4 into YSG2 strain and analyzed as in Figure 2A. To induce the *GCN4-lacZ* expression, the transformants grown at the minimal media for 2 h after dilution were treated with 10 mM 3-AT for 6 h. The black bar represents the intercistronic sequence between uORF4 and *GCN4* and the gray bars represent the (CAA)*n* insertions.

A simple comparison of the induction potential of uORF1 + 4 and uORF2 + 4 constructs under starvation conditions induced by the addition of 3-aminotriazole (3-AT; an inhibitor of histidine biosynthetic genes) shows that whereas the former arrangement induces the *GCN4-lacZ* expression by ∼5.2-fold, the latter shows ∼3.6-fold induction (Figure 6). This clearly demonstrates that uORF2 operates, at least in this minimalistic system, in the similar fashion as uORF1, reaching significant 70% of its induction potential. The most likely explanation for the ∼30% difference in induction potentials between uORFs 1 and 2 could be the shorter distance between uORF2 and the *GCN4-lacZ* main ORF compared with that between uORF1 and the *GCN4-lacZ*. This would—according to the accepted logic of the *GCN4* translational control mechanism—provide the post-termination ribosomes scanning downstream from uORF2 versus uORF1 less time to reacquire the TC before reaching the *GCN4* start codon under starvation conditions, when the pool of the TCs is decreased. Consequently, the *GCN4* start codon would be bypassed. To test this, we increased the distance between uORF2 and *GCN4-lacZ* to make it equal to the distance between uORF1 and *GCN4-lacZ*. One way to do this was to take a sequence of a defined length from the intercistronic region between uORF4 and the *GCN4* main ORF and insert it in front of uORF4 (Figure 6, construct ORF2-ins68 + 4) to avoid introduction of potential side effects of randomly inserted sequences on the REI process—the similar strategy was successfully used in the past (26,27). In an alternative approach, uORF1 and its 5′ and 3′ flanking sequences (segments A–D) were replaced in the 1111 + 4 construct by the corresponding sequences of uORF2 (Supplementary Figure S3, construct 2222 + 4). Contrary to our prediction, in both cases these length manipulations decreased (to ∼50%) but not increased the induction of *GCN4-lacZ* expression under starvation conditions (Figure 6 and Supplementary Figure S3). Interestingly, gradual shortening of the artificially increased distance between uORF2 and *GCN4-lacZ* in the ORF2-ins68 + 4 construct through 47 nt (ORF2-ins(CAA)47 + 4) down to 26 nt (ORF2-ins(CAA)26 + 4) gradually increased the levels of the *GCN4-lacZ* induction by 3-AT (Figure 6). (Note that in the latter two constructs the unstructured sequence of CAA trinucleotide repeats was used to modify their length instead of the above defined uORF4–*GCN4-lacZ* intercistronic sequence.) These surprising findings could mean that perhaps the induction activity of uORF2 under starvation condition is at its maximum in its native arrangement (corresponding to 70% of the uORF1 activity), and for some yet to be uncovered reasons it strictly requires the natural distance between uORF2 and uORF4 to be kept. In support, matching the distance between uORF2 and *GCN4-lacZ* with that of uORF1 and *GCN4-lacZ* by doubling the 68 nt long segment in the uORF4–*GCN4-lacZ* intercistronic region while preserving the uORF2 – uORF4 natural distance (Figure 6, construct ORF2 + 4-ins68) produced virtually identical β-galactosidase activity to that of the ‘wt’ uORF2 + 4 construct; i.e. ∼71% of the uORF1 + 4 construct. Furthermore, moving uORF1 and its 5′ and 3′ flanking sequences into the position of uORF2 by deleting 68 nt downstream of uORF1 strikingly increased the induction potential of uORF1 in the uORF1-Δ68 + 4 construct by ∼20% compared with the original uORF1 + 4 construct (Figure 6). Together these results surprisingly imply that the distance of uORF2 from uORF4 is optimal for the maximal REI potential not only for uORF2 but also for uORF1. This suggests that (i) the uORF2 – *GCN4-lacZ* distance is long enough to provide the post-termination ribosomes scanning downstream from uORF2 sufficient time to reacquire the TC before reaching the *GCN4* start codon under starvation conditions, and (2) that there is something very specific about the general reinitiation mechanism with respect to mutual positioning of individual uORFs that we still do not understand (see ‘Discussion’ section).

### Fail-safe mechanism of *GCN4* translational control

**Figure 7.**
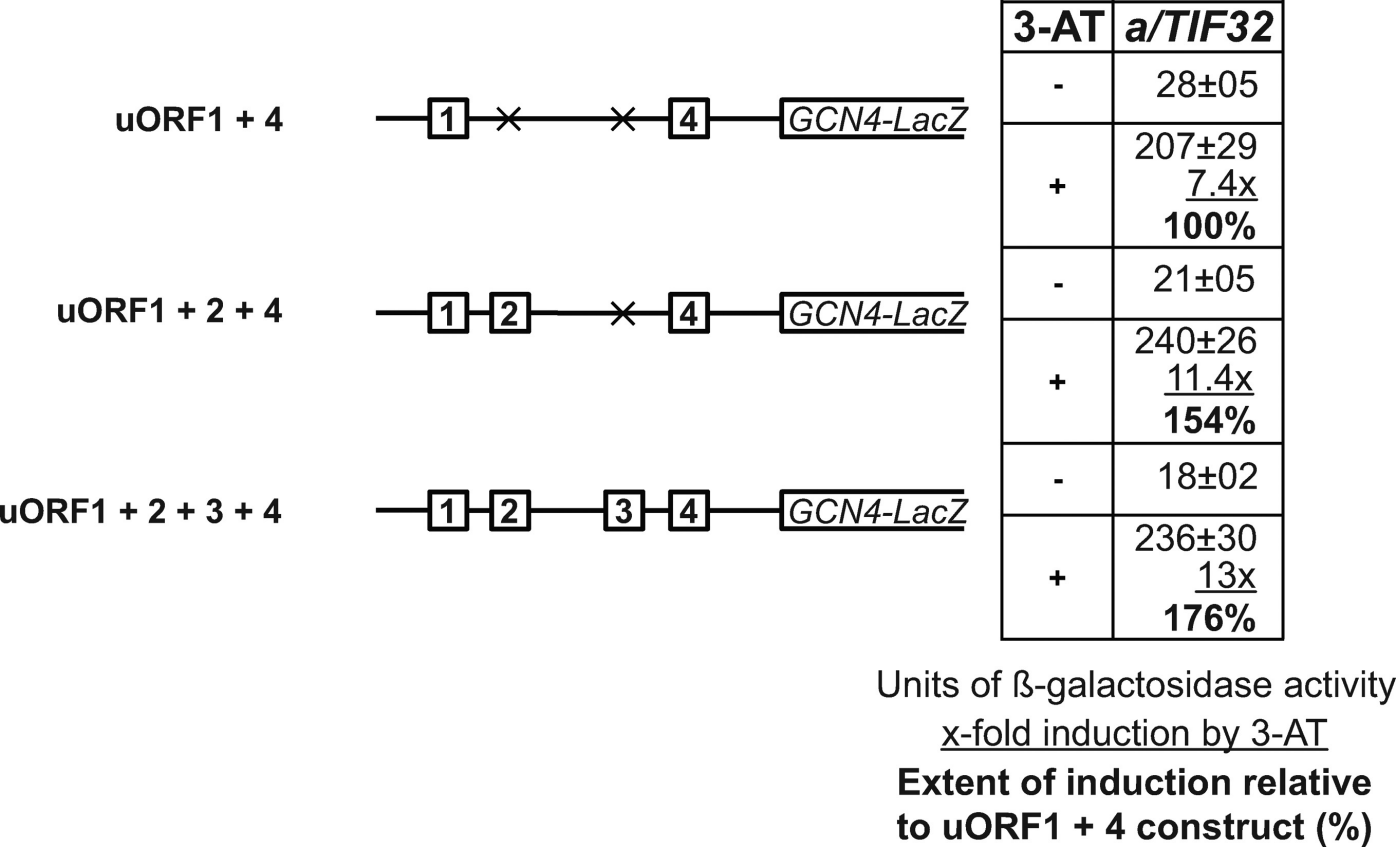
uORF2 backs up the role of uORF1 in the *GCN4* translational control. The wt *GCN4-lacZ* construct carrying all four uORFs was modified as depicted in the schematics to the left of the table. The resulting mutant constructs were introduced together with the wt construct into YSG2 strain and analyzed as in Figure 6.

We next asked the question as to why two consecutive REI-permissive uORFs occur in the same mRNA leader, if only one is sufficient to promote translational regulation of *GCN4* expression to a reasonable degree. The working hypothesis was that they perhaps create some sort of a back-up system preventing miss-regulation of *GCN4* expression in situations when the start codon of the first uORF is skipped by scanning pre-initiation complexes. To test this, we assayed the induction potential of the wt uORF1 + 2 + 3 + 4 construct side by side with the minimalistic uORF1 + 4 and semi-minimalistic uORF1 + 2 + 4 constructs under starvation condition. The rational was that if uORF2 backs up the role of uORF1 in the *GCN4* translational control as some sort of a fail-safe guardian, the induction potential of uORF1 + 2 + 4 should be higher than that of uORF1 + 4 but not higher than uORF1 + 2 + 3 + 4. As shown in Figure 7, this is exactly what was observed. It should be noted, however, that the overall increase in the induction potential of uORF1 + 2 + 4 and uORF1 + 2 + 3 + 4 constructs by 54 and 76%, respectively, can be partially ascribed to decreased basal levels that these two constructs display under non-starvation conditions. This fact on its own already clearly illustrates that having uORF1 backed up by uORF2 makes the whole regulatory system more stringent. When we disregard the basal levels and compare only the absolute values of induction, both uORF1 + 2 + 4 and uORF1 + 2 + 3 + 4 constructs show ∼115% of the uORF1 + 4 activity. This small but significant increase in the REI activity under nutrient deplete conditions then most likely reflects the real amount of pre-initiation complexes that naturally skipped the uORF1 AUG but got caught by uORF2 to expand the REI capacity of the entire regulatory system; i.e. to increase the number of post-termination 40S ribosomes that have already resumed scanning after translating uORF1 toward the *GCN4* AUG start site.

## DISCUSSION

Regulation of yeast *GCN4* expression represents undoubtedly the best studied model for the uORF-mediated translational reinitiation. The *GCN4* mRNA leader contains together four short uORFs, however, only the first one—uORF1—has been described as REI-permissive. In this work, we demonstrate that, in contrast to the current model, uORF2 is the second REI permissive uORF within the *GCN4* mRNA leader, with nearly as efficient REI potential as is that of uORF1. Moreover, we show that in striking analogy with uORF1 of *GCN4* and uORF of *YAP1*, the molecular mechanism by which uORF2 promotes REI also depends on the presence of specific upstream REI promoting elements and the a/TIF32-NTD. Contrary to uORF1 operating with four RPEs, however, uORF2 relies only on two *cis*-acting sequences: (i) the eIF3-independent RPE ii. shared with uORF1, and (ii) its own, specific RPE v. working via its functional interaction with the a/TIF32-NTD. This strongly suggests that the underlying REI mechanism in yeasts is highly conserved. Furthermore, the existence of the second REI-permissive uORF in the *GCN4* mRNA leader evokes the idea that its consecutive REI-permissive and REI-nonpermissive uORFs (1 and 2 versus 3 and 4) were doubled to create a fail-safe mechanisms ensuring stringent regulation of its expression under non-starvation versus starvation conditions. This view thus markedly overwrites our current perception of this longstanding, exemplary reinitiation model of the *GCN4* translation control.

### Conserved *cis*-acting elements promoting reinitiation in yeast

RPE ii. of uORF1 was previously identified as a 22 nt-long bulged stem with a small apical loop of 3 nt, the function of which does not depend on eIF3 (12). Strikingly, here we found that this particular element is also used by uORF2. Interestingly, although the distance between RPE ii. and the uORF2 coding region is approximately twice the length of the RPE ii.—uORF1 distance, the contribution of RPE ii. to the overall REI activity is higher for uORF2 (∼70%) (Figure 4) than for uORF1 (∼40%) (12). It is conceivable that this difference in REI activities in the absence of RPE ii. is caused by another eIF3-independent RPE in the uORF1 leader—RPE iii.—that is not utilized by uORF2, which could in case of uORF1 partially compensate for the missing RPE ii. activity. However, taken into account its relatively modest contribution to the overall REI activity of uORF1 (∼20%) (12), a more likely explanation is that RPE ii. is brought closer to the termination complex via mRNA looping that is more effective for ribosomes terminating on uORF2 thanks to its longer distance from RPE ii. compared with uORF1. The RPE ii. brought to the vicinity of the post-termination 40S subunit could make a direct contact with a small ribosomal protein(s) or some ribosome-associated co-factor(s) to provide the stabilization effect for the 40S subunit necessary for subsequent resumption of scanning. It is less likely that it would interact with 18S rRNA, like in the case of termination upstream ribosome binding site (TURBS) promoting REI after long ORF in bicistronic subgenomic caliciviral mRNAs (28), because RPE ii. is a double stranded stem with only three free nt in the apical loop. In any case, this either direct or bridged interaction with the termination complex would be thanks to mRNA looping more efficient in case of uORF2 than uORF1.

The second and last 5′ UTR element (RPE v.) needed specifically for uORF2-promoted REI was identified due to its sequence homology with the 3′ half of the uORF1-specific RPE i. Both of these elements are AU-rich and their function depends on the interaction with the a/TIF32-NTD. Even though we still cannot tell for sure if this interaction is direct or not, there are several observations supporting the former option. When ribosomes terminate on either uORF1 or 2, RPE i. or RPE v., respectively, occur close to the mRNA exit channel, where the NTD of a/TIF32 sits (10,21,22). Consistently, the a subunit of mammalian eIF3 was also proposed to occur in this area (29). Thus, the shared sequence motif between RPEs i. and v. [(UUA)_2_UC/UAG] could indeed specifically bind to the NTD of a/TIF32, which was only recently shown to carry patches of positive charge on its surface and to be capable of RNA binding (30). In this aspect, the REI promoted by these two RPEs could bear a certain resemblance with the aforementioned caliciviral TURBS that besides 18S rRNA also interacts with eIF3 (31). To understand the nature of the interaction between the a/TIF32-NTD and RPEs i. and v. is a pressing task that is being extensively studied in our laboratory.

Besides these obvious similarities, it should also be mentioned that the exact distance between the 3′ end of RPE i. and AUG of uORF1 (30 nt) and RPE v. and AUG of uORF2 (18 nt) differs by 12 nt. Thus, the efficiency with which these two elements are contacted by the 40S-bound eIF3 may also differ, even though their individual contributions to the overall REI activity of either uORF1 or uORF2 are similar (between 30 and 40%) (Figure 5) (12). There is, however, one important difference. Contrary to RPE v., the uORF1-specific RPE i. requires another a/TIF32-dependent element, structured RPE iv., with which it works in close synergy (12). It is therefore conceivable that this close cooperation between RPE i. and RPE iv. might be required to overcome the constraint of a longer distance of RPE i. from the uORF1 start codon. On the other hand, activity of another REI permissive uORF in the *YAP1* mRNA leader seems to rely solely on the RPE iv.-like feature (no sequence similarities with *GCN4* RPE i. were found upstream of *YAP1* uORF) suggesting that even this type of a structured *cis-*acting element might work on its own, well, in co-operation with the a/TIF32-NTD (12).

**Figure 8.**
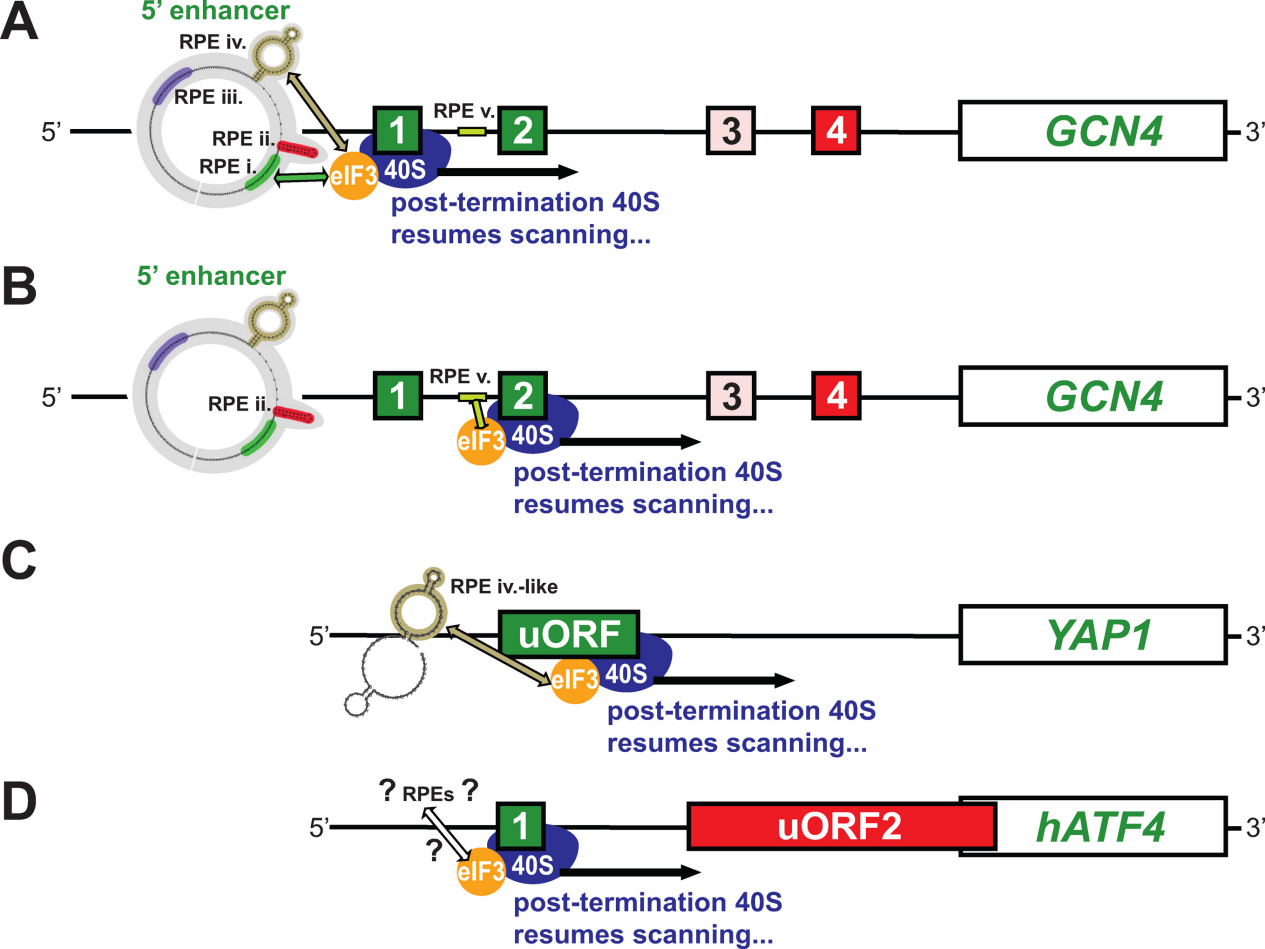
Schematics summarizing unifying features of the molecular mechanism utilized by uORF1 (**A**) and uORF2 (**B**) of *GCN4* and uORF (**C**) of *YAP1* ensuring high efficiency of REI. Functional interactions between the eIF3-dependent REI-promoting elements (RPEs) and the NTD of a/TIF32 are symbolized by arrows. (**D**) Schematic hypothetically proposing a similar molecular mechanism of REI on uORF1 of the *GCN4* functional homologue in higher eukaryotes *ATF4*.

Our current knowledge of a molecular mechanism ensuring efficient REI is summarized in Figure 8. Successful resumption of scanning of post-termination 40S ribosomal subunits on short uORFs for REI downstream is determined by two major factors: (i) specific 5′ REI promoting elements and (ii) functional interaction of some of them with a/TIF32-NTD of eIF3. In detail, resumption of scanning promoted by *GCN4* uORF1 requires four RPEs (RPE i.–iv.), two of which (RPE i. and RPE iv.) act synergistically by interacting with a/TIF32 (Figure 8A). The *GCN4* uORF2 relies on the activity of only two RPEs, the RPE ii. of uORF1 and the eIF3-dependent RPE v. with significant sequence similarity to RPE i. of uORF1 (Figure 8B). It seems that the proximity of RPE i. and RPE v. to AUGs of both uORFs is important for their functions most probably due to their contact with 40S-bound eIF3. Conversely, RPE ii. appears to function in a distance-independent manner, at least to a certain extent. Finally, the *YAP1* uORF seems to utilize only one element, which is structurally similar to RPE iv. of uORF1 and operates in the a/TIF32-NTD-dependent manner (Figure 8C). Taking together, the presented schemes nicely illustrate the common features of the REI process strongly suggesting that the basic principles of this important regulatory mechanism of gene expression are, at least among yeasts, very well conserved. Now it will be intriguing to expand our knowledge to higher eukaryotes and investigate whether or not the similar features determine the REI permissiveness also for those genes, the mRNAs of which are subjected to this type of regulation, such as the functional homologue of yeast *GCN4*, mammalian *ATF4* (Figure 8D).

### Fail-safe mechanism of *GCN4* translational control

By using a minimalistic regulatory system containing only uORF1 and uORF4 it was shown that full capacity of *GCN4* induction under starvation conditions is strongly dependent on the relative distance between these two uORFs, and between uORF1 and *GCN4* (15). Increasing the distance between uORF1 and uORF4 reduced the basal level of *GCN4* expression under non-starvation conditions and markedly compromised induction of *GCN4* expression under starvation conditions, the latter of which was attributed to the increased probability of rescanning 40S ribosomes to rebind TCs before reaching uORF4 (26). Decreasing the uORF1–uORF4 spacing by bringing uORF1 a lot closer to *GCN4* also led to reduction in *GCN4* expression under starvation conditions, this time because the probability of rebinding TCs before reaching *GCN4* was decreased (27). We show here that a similar minimalistic system composed of only uORF2 and uORF4 is also capable of relatively efficient *GCN4* regulation (Figure 6). In analogy with the uORF1 + 4 system, the uORF2 + 4 system reacts similarly to increasing the distance between these two uORFs as it reduces the basal level of *GCN4* expression under non-starvation conditions and the extent of *GCN4* induction under starvation conditions. Another common characteristic of both systems is that increasing the uORF4–*GCN4* spacing shows a little to no effect (Figure 6) (26). We also demonstrated that shortening the distance between uORF1 and uORF4 by only 68 nt [i.e. by roughly one half of what was examined before (27)] did not decrease but surprisingly increased the extent of *GCN4* induction under starvation conditions (Figure 6). Together our results suggest that the ‘spacing’ requirements of both REI-permissive uORFs with respect to uORF4 and *GCN4* play an important role in maintaining their proper responsiveness to changing TC levels under nutrient replete or deplete conditions, with the position of uORF2 serving as optimal for the maximal REI potential of both uORFs. The true nature of this role remains to be elucidated because the logic that it is all determined by the TC levels simply does not apply here.

**Figure 9.**
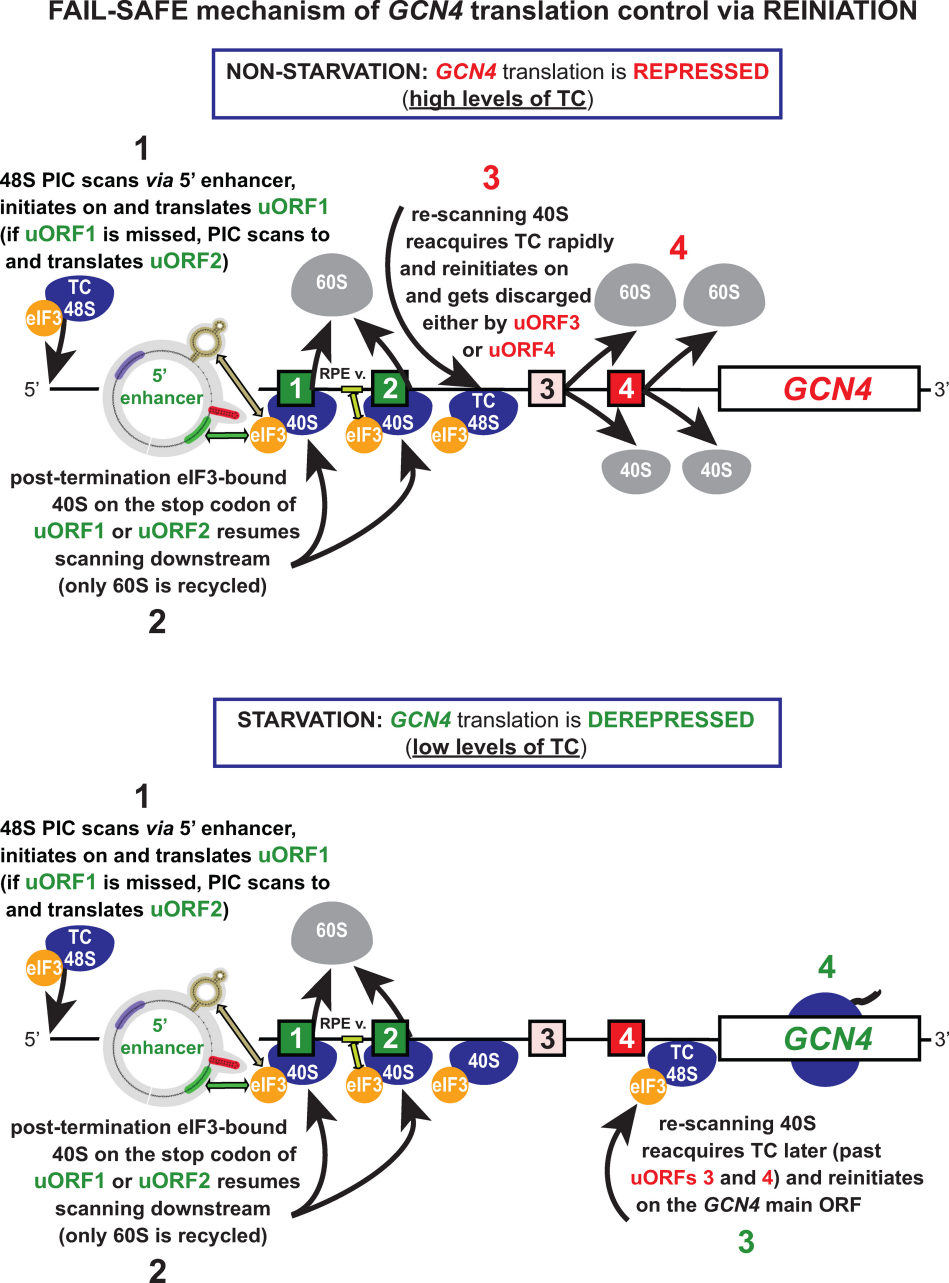
Model of the ‘Fail-safe mechanism’ of *GCN4* translational control. Schematic of the *GCN4* mRNA leader showing distribution of all four short uORFs, locations of the uORF1-specific and uORF2-specific RPEs, 40S-bound eIF3, and the description of the ‘Fail-safe mechanism’ of the *GCN4* translational control. Upper panel models the events on the *GCN4* mRNA leader occuring under non-starvation conditions with abundant TC levels—‘*GCN4*-expression repressed state’, the lower panel illustrates the steps that take place under starvation condition with limited supply of the TC—‘*GCN4*-expression derepressed state’ (see text for further details).

Presence of multiple uORFs in the mRNA 5′ leader is widely considered to impose a strong inhibitory, rather than stimulatory, effect on translation. Hence, it was not overly surprising that only the first uORF from the *GCN4* mRNA was considered to be REI-permissive. With respect to our novel findings, however, the question emerges as to what is the natural selection advantage to keep or insert the second, almost equally REI-permissive uORF in the single mRNA leader? Especially so, when only two uORFs—one REI-permissive and the other non-permissive—suffice for a reasonable degree of regulation of *GCN4* expression (15,18). Even though the AUG start codon of uORF1 was shown to serve as an efficient translational start site (32) (uORF1 shows only ∼3% of leaky scanning (10)), the only logical explanation could be that the arrangement of two consecutive REI-permissive uORFs creates some sort of a back-up system as experimentally demonstrated in Figure 7. This system most likely prevents miss-regulation of *GCN4* expression in situations when the start codon of the first uORF is skipped by scanning pre-initiation complexes. This could happen for example under specific stress conditions, when *GCN4* expression needs to be derepressed via phosphorylation of eIF2 by GCN2 kinase, and the stress factor at the same time also increases the frequency of leaky scanning (amino acid starvation is by far not the only one trigger of *GCN4* expression (14)). In support of this idea, stress-induced phosphorylation of eIF2 was shown not only to reduce the TC levels, but also to facilitate ribosomal bypass of a single inhibitory uORF to enhance *CHOP* translation under endoplasmic reticulum stress in mammalian cells (33). Furthermore, an increase in leaky scanning of AUGs of short regulatory uORFs upon eIF2 phosphorylation was also seen in other stress induced genes (34,35). Thus, the high REI permissiveness of uORF2 might be seen as an effective fail-save mechanism (Figure 9 and Supplementary Figure S4), which has evolved or been preserved over time, because stringent control over *GCN4* translation is a critical demand of yeast cells under various stress conditions. In fact, this system in principle repeats itself immediately downstream of uORF2 in two consecutive REI-non-permissive uORFs, uORF3 and uORF4, both ensuring the tightness of the entire regulatory mechanism (Figure 9 and Supplementary Figure S4). Importantly, presence of three or more uORFs preceding the *GCN4* gene can be also found among all hemiascomycetal yeast species, and four uORFs with similar positioning within the 5′ mRNA leader of *GCN4* are specifically conserved among those species that diverged after the whole genome duplication (36).

Finally, a direct comparison of REI permissiveness of uORF3 and uORF4 indicates that uORF3 is approximately four times less inhibitory than the super-inhibitory uORF4 (Figure 2). This super-inhibitory effect was attributed to the GC-rich sequences immediately following the stop codon of uORF4, because the replacement of the AU-rich 3′ sequences of uORF1 with those of uORF4 was sufficient to convert uORF1 into a strong inhibitor of REI as well (18). Since deletion of all RPEs in the uORF3-only construct had no effect on its already low REI activity (Figure 3B), and since its 3′ sequences are also AU-rich (12), we propose that uORF3 probably represents a typical yeast uORF with no specific characteristics that would render it either super-permissive or super-inhibitory for REI. This implies that in order to create such a sophisticated regulatory mechanism such as that of *GCN4*, the nature invented 5′ elements—the RPEs—as well as the 3′ feature—GC-rich sequences, that either dramatically increase the REI potential of short uORFs (in case of *GCN4* uORFs 1 and 2, or *YAP1* uORF) or decrease it (in case of *GCN4* uORF4).

## SUPPLEMENTARY DATA

Supplementary Data are available at NAR Online, including [10,12,15,18–20,24].

SUPPLEMENTARY DATA
