# Supplementary material for: Fail-safe mechanism of GCN4 translational control—uORF2 promotes reinitiation by analogous mechanism to uORF1 and thus secures its key role in GCN4 expression
Source: Nucleic Acids Res. 2014 Mar 12;42(9):5880–93. doi: 10.1093/nar/gku204 (PMC4027193; doi:10.1093/nar/gku204)
Supplement: SUPPLEMENTARY DATA [file supp_42_9_5880__index.html]

Fail-safe mechanism of GCN4 translational control—uORF2 promotes reinitiation by analogous mechanism to uORF1 and thus secures its key role in GCN4 expression — SUPPLEMENTARY DATA 

# Fail-safe mechanism of *GCN4* translational control—uORF2 promotes reinitiation by analogous mechanism to uORF1 and thus secures its key role in *GCN4* expression

## SUPPLEMENTARY DATA

**Files in this Data Supplement:**

- Supplemental Figures
