# Supplementary material for: Fail-safe mechanism of GCN4 translational control—uORF2 promotes reinitiation by analogous mechanism to uORF1 and thus secures its key role in GCN4 expression
Source: Nucleic Acids Res. 2014 Mar 12;42(9):5880–93. doi: 10.1093/nar/gku204 (PMC4027193; doi:10.1093/nar/gku204)
Supplement: SUPPLEMENTARY DATA [file supp_gku204_nar-00040-v-2014-File011.pdf]

SUPPLEMENTARY DATA

MATERIALS AND METHODS

Plasmid construction

Plasmids pSG60, pSG61 and pSG62 were created with the help of QuikChange II XL Site-Directed Mutagenesis Kit (Agilent Technologies) according to the manufacturer´s recommendations. For pSG60 and pSG62 the oligonucleotides SG9 and SG10 and for pSG61 the oligonucleotides SG11 and SG12 were used, respectively. Synthesis of pSG60 and pSG61 was templated by p227, and p226 served as a template for pSG62.

To construct pSG66 and pSG67 by fusion PCR, the following pairs of primers and templates were used: (i) BS144 / SG13 and pM23; (ii) SG14 / SG15 and pSG60; and (iii) SG16 / SG17 and pSG61. For pSG66 the PCR products obtained from (i) and (ii) were mixed in a 1:1 ratio as templates for a subsequent PCR amplification with primers BS144 and SG15. Similarly, for pSG67 the products of (i) and (iii) were combined and primers BS144 and SG17 were added. The resulting PCR products were digested with *Sal*I and *Bgl*II and ligated into *Sal*I-*Bgl*II cleaved pM23.

Plasmids pSG73 and pSG74 were produced by fusion PCR. First, the combinations of primers BS144 / SG22 and SG20 / SG21 were used in PCR reactions templated by pSG66 for pSG73 and by pSG67 for pSG74. The PCR products obtained from corresponding reactions were used in a 1:1 ratio as templates for a third PCR amplification with primers BS144 and SG21. The resulting PCR products were digested with *Sal*I and *Cla*I and ligated into *Sal*I-*Cla*I cleaved pSG66 or pSG67, respectively.

All plasmids listed below were created with the help of fusion PCR using the indicated two pairs of primers and DNA templates: for pSG69 (i) BS144 / SG18 and pSG60 as template and (ii) SG19/ BS126 and pSG60; for pSG71 (i) BS144 / SG18 and pSG61 and (ii) SG19 / BS126 and pSG61; for pSG75 (i) BS144 / SG26 and pSG60 and (ii) SG25 / BS126 and pSG60; for pSG76 (i) BS144 / SG28 and pSG73 and (ii) SG27 / BS126 and pSG73; for pSG77 (i) BS144 / SG29 an pSG60 and (ii) SG27 / BS126 and pSG73; for pSG78 (i) BS144 / SG30 and pSG62 and (ii) SG31 / BS126 and pSG62; for pSG86 (i) BS144 / VM46 and pSG60 and (ii) VM45 / BS126 and pSG60; for pSG100 (i) BS144 / VM46 and pSG77 and (ii) VM45 / BS126 and pSG77; for pSG88 (i) BS144 / VM8 and pSG60 and (ii) VM7 / BS126 and pSG60; for pSG102 (i) BS144 / VM8 and pSG77 and (ii) VM7 / BS126 and pSG77; for pSG89 (i) BS144 / VM72 and pSG73 and (ii) VM73 / BS126 and pSG73; for pSG91 (i) BS144 / VM72 and pSG76 and (ii) VM73 / BS126 and pSG60; for pSG90 (i) BS144 / VM40 and pSG73 and (ii) VM38 / BS126 and pSG73; for pSG93 (i) BS144 / VM40 and pSG60 and (ii) VM38 / BS126 and pSG60; for pSG138 (i) BS144 / SG85 and pSG60 and (ii) SG86 / BS126 and pSG60; for pSG141 (i) BS144 / SG85 and pSG93 and (ii) SG86 / BS126 and pSG93; for pSG176 and pSG178 (i) BS144 / SG47 and pSG62 and (ii) SG67 / BS126 and pSG62, respectively; for pSG237 (i) BS144 / SG195 and

p196 and (ii) SG196 / BS126 and p196; for pSG244 (i) BS144 / SG10 and p196 and (ii) SG9 / BS126 and p196. Thus obtained PCR products were used in a 1:1 ratio as templates for a third PCR amplification with primers BS144 and BS126. The resulting PCR products were digested with *Sal*I and *Bst*EII and ligated into *Sal*I-*Bst*EII cleaved p209, pSG60, pSG62 or pSG73. Plasmids pSG176 and pSG178 were originally designed to contain a 68 nt insert of CAA repeats; truncations to 26 and 47 nt were most probably generated by recombination during plasmid replication in bacterial cells.

Plasmid pSG179 was created using fusion PCR with pSG62 serving as a template and following combinations of primers: (i) SG25 / SG111 and (ii) SG110 / SG21. Primers SG25 and SG21 were then used in the third reaction with a 1:1 ratio of PCR products from the first and second reactions as templates. The resulting PCR product was digested with *Bst*EII and *Cla*I and inserted into *Bst*EII-*Cla*I cut pSG62.

REFERENCES

12. Munzarová, V., Pánek, J., Gunišová, S., Dányi, I., Szamecz, B. and Valášek, L.S. (2011) Translation Reinitiation Relies on the Interaction between eIF3a/TIF32 and Progressively Folded cis-Acting mRNA Elements Preceding Short uORFs. *PLoS Genet*, **7**, e1002137.
24. Mueller, P.P., Harashima, S. and Hinnebusch, A.G. (1987) A segment of *GCN4* mRNA containing the upstream AUG codons confers translational control upon a heterologous yeast transcript. *Proc Natl Acad Sci USA*, **84**, 2863-2867.
19. Grant, C.M. and Hinnebusch, A.G. (1994) Effect of sequence context at stop codons on efficiency of reinitiation in *GCN4* translational control. *Mol Cell Biol*, **14**, 606-618.
15. Mueller, P.P. and Hinnebusch, A.G. (1986) Multiple upstream AUG codons mediate translational control of *GCN4*. *Cell*, **45**, 201-207.
18. Miller, P.F. and Hinnebusch, A.G. (1989) Sequences that surround the stop codons of upstream open reading frames in *GCN4* mRNA determine their distinct functions in translational control. *Genes and Development*, **3**, 1217-1225.
10. Szamecz, B., Rutkai, E., Cuchalova, L., Munzarova, V., Herrmannova, A., Nielsen, K.H., Burela, L., Hinnebusch, A.G. and Valášek, L. (2008) eIF3a cooperates with sequences 5' of uORF1 to promote resumption of scanning by post-termination ribosomes for reinitiation on GCN4 mRNA. *Genes Dev*, **22**, 2414-2425.
20. Grant, C.M., Miller, P.F. and Hinnebusch, A.G. (1995) Sequences 5' of the first upstream open reading frame in *GCN4* mRNA are required for efficient translational reinitiation. *Nuc Acids Res*, **23**, 3980-3988.

SUPPLEMENTARY FIGURES AND FIGURE LEGENDS

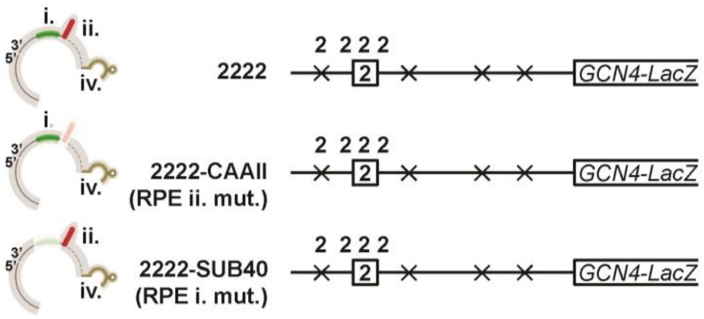

	a/TIF32	-Δ*8*	*-Box6+17*
2222	517±30 **100%**	236±40 **100%**	366±21 **100%**
2222-CAAII (RPE ii. mut.)	332±07 **64%**	103±08 **44%**	183±18 **50%**
2222-SUB40 (RPE i. mut.)	555±16 **107%**	212±21 **90%**	304±17 **83%**

Units of ß-galactosidase activity
Activity relative to 2222 construct in corresp. strains (%)

Supplementary Figure S1. The RPE ii. of uORF1 serves as a common, eIF3-independent REI-promoting element for both uORF1 and uORF2. The same as in Figure 4 except that the previously identified substitutions of uORF1-specific RPE ii. (CAAII) and RPE i. (SUB40) were individually introduced into the 2222 construct and analyzed.

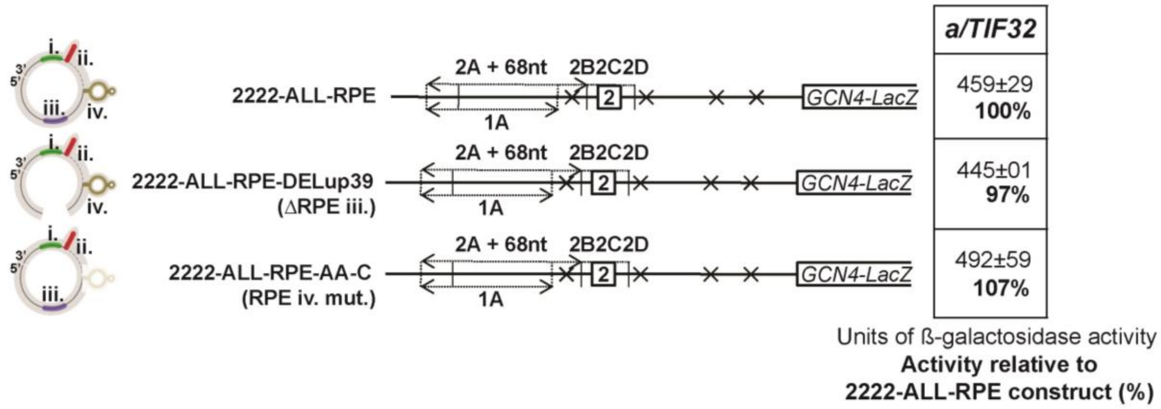

Supplementary Figure S2. The RPEs iii. and iv. of uORF1 are not utilized by the uORF2-specific REI promoting mechanism. The same as in Figure 4 except that the previously identified mutations of uORF1-specific RPE iii. (ΔRPE iii.) or RPE iv. (AA-C) were individually introduced into the 2222-ALL-RPE construct (described in Figure 3A) and analyzed.

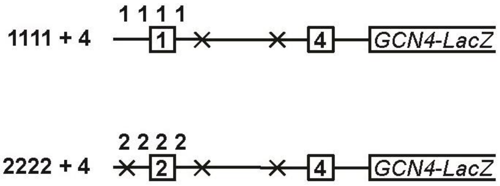

3-AT	*a/TIF32*
-	64±01
+	421±68<br>6.6x<br>**100%**
-	24±02
+	81±03<br>3.4x<br>**51%**

Units of ß-galactosidase activity
x-fold induction by 3-AT
Extent of induction relative to 1111 + 4 construct (%)

Supplementary Figure S3. uORF2 operates in the minimalistic *GCN4* translational control system composed of only uORFs 2 and 4 in the similar fashion as uORF1. The same as in Figure 6 except that the construct carrying uORF2 at the position of uORF1 combined with uORF4 was analyzed together with the construct carrying uORF1 and uORF4 in their native positions.

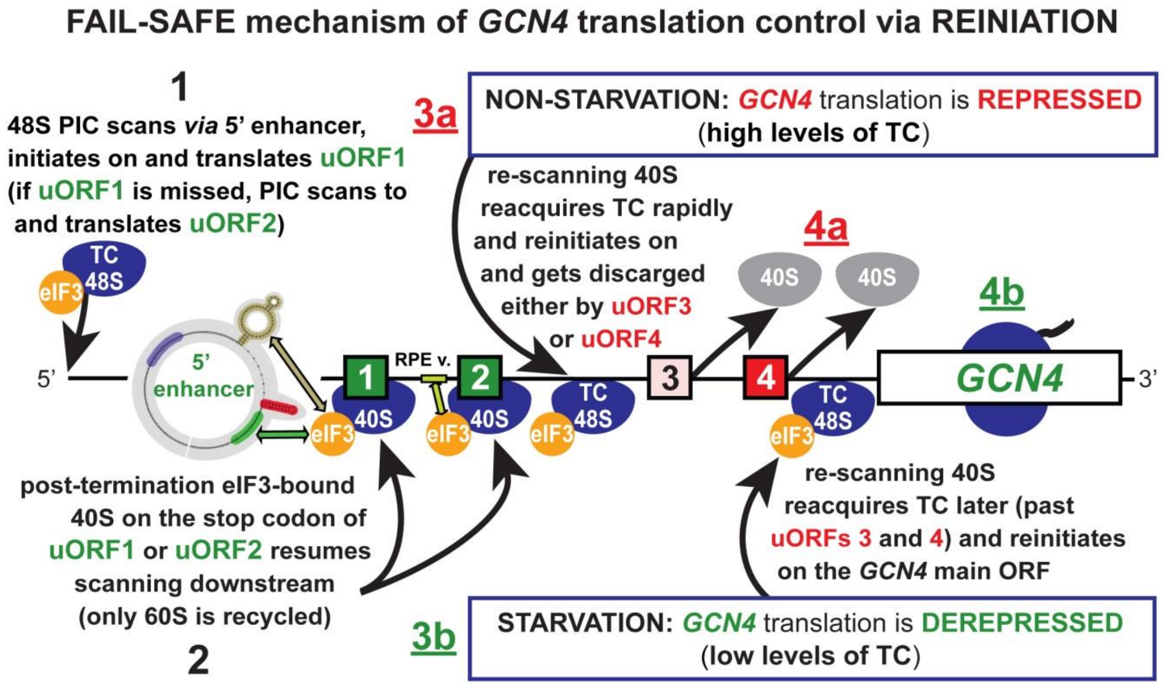

Supplementary Figure S4. Simplified model of the "Fail-safe mechanism" of *GCN4* Translational Control. Schematic of the *GCN4* mRNA leader showing distribution of all four short uORFs (REI-permissive uORFs 1 and 2 are labeled green; REI-non-permissive uORFs 3 and 4 are labeled in light and dark red, respectively), locations of the uORF1-specific and uORF2-specific RPEs, 40S-bound eIF3, and the description of the "Fail-Safe mechanism" of the *GCN4* translation control. The 3a and 4a "*GCN4*-expression repressed" steps take places under non-starvation conditions with abundant ternary complex (TC) levels, whereas the 3b and 4b "*GCN4*-expression derepressed" steps occur under starvation condition with limited supply of the TC (see text for further details).

SUPPLEMENTARY TABLES

Supplementary Table S1. Yeast strains used in this study.

**Strain**	**Genotype**	**Source or reference**
YSG2 [a]	*MAT*a *leu2-3, -112 ura3-52 trp1Δ gcn2Δ tif32Δ URA3::GCN2 ura3* (YCp-a/TIF32-His-L)	(12)
YSG15 [a]	*MAT*a *leu2-3, -112 ura3-52 trp1Δ gcn2Δ tif32Δ URA3::GCN2 ura3* (YCp-a/tif32-Δ8-His-L)	(12)
YSG38 [a]	*MAT*a *leu2-3, -112 ura3-52 trp1Δ gcn2Δ tif32Δ URA3::GCN2 ura3* (YCp-a/tif32-Box6+17-His-L)	(12)

[a] Isogenic strains.

Supplementary Table S2. Plasmids used in this study.

**Plasmid**	**Description**	**Source of reference**
p227 (uORFless)	low copy URA3 vector containing uORFless *GCN4* leader created by point mutations in uORF1 (*Hind*III), uORF2 (*Eco*RI), uORF3 (*Kpn*I) and uORF4 (*Bgl*II)	(24)
p209 (uORF1-only)	low copy URA3 vector containing uORF1 only at its original position	(19)
p226 (uORF4-only)	low copy URA3 vector containing uORF4 only at its original position	(24)
pSG60 (uORF2-only)	low copy URA3 vector containing uORF2 only at its original position	This study
pSG61 (uORF3-only)	low copy URA3 vector containing uORF3 only at its original position	This study
p196 (uORF1+4)	low copy URA3 vector containing uORF1 and uORF4 only at their original positions	(15)
pSG62 (uORF2+4)	low copy URA3 vector containing uORF2 and uORF4 only at their original positions	This study
pM23 (1111+4)	low copy URA3 vector containing uORF1 and uORF4 only at their original positions, with *Hind*III and *Bgl*II restriction sites upstream and downstream from uORF1, respectively	(18)
pSG66 (2222+4)	low copy URA3 vector containing uORF1 and uORF4 only at their original positions , with *Bgl*II restriction site downstream from uORF1, uORF1's 5' flanking sequences (nt -181 to -1), the coding region, and the 3' flanking sequences (25 nt beyond the stop codon) were replaced by the corresponding sequences of uORF2	This study
pSG67 (3333+4)	low copy URA3 vector containing uORF1 and uORF4 only at their original positions , with *Bgl*II restriction site downstream from uORF1, uORF1's 5' flanking sequences (nt -181 to -1), the coding region, and the 3' flanking sequences (25 nt beyond the stop codon) were replaced by the	This study

	corresponding sequences of uORF3	
pBS63 (uORF1-only-Δ160)	low copy URA3 vector containing uORF1 only at its original position with 160 nt deletion in its 5' UTR (from -181 to -21)	(10)
pSG69 (uORF2-only- Δ160)	low copy URA3 vector containing uORF2 only at its original position with 160 nt deletion in 5' UTR of uORF1 (from -181 to -21)	This study
pSG71 (uORF3-only- Δ160)	low copy URA3 vector containing uORF3 only at its original position with 160 nt deletion in 5' UTR of uORF1 (from -181 to -21)	This study
pM128 (1111)	low copy URA3 vector containing uORF1 only at its original position, with *Hind*III and *Bgl*II restriction sites upstream and downstream of uORF1, respectively	(20)
pVM35 (4444)	low copy URA3 vector containing uORF1 only at its original position, with *Hind*III and *Bgl*II restriction sites upstream and downstream of uORF1, respectively; uORF1's 5' flanking sequences (nt -181 to -1), the coding region, and the 3' flanking sequences (25 nt beyond the stop codon) were replaced by the corresponding sequences of uORF4	(12)
pSG73 (2222)	low copy URA3 vector containing uORF1 only at its original position, with *Bgl*II restriction site downstream of uORF1, uORF1's 5' flanking sequences (nt -181 to -1), the coding region, and the 3' flanking sequences (25 nt beyond the stop codon) were replaced by the corresponding sequences of uORF2	This study
pSG74 (3333)	low copy URA3 vector containing uORF1 only at its original position, with *Bgl*II restriction site downstream of uORF1, uORF1's 5' flanking sequences (nt -181 to -1), the coding region, and the 3' flanking sequences (25 nt beyond the stop codon) were replaced by the corresponding sequences of uORF2	This study
pSG75 (uORF2-only- Δ233)	low copy URA3 vector containing uORF2 only at its original position with 233 nt deletion in its 5' UTR (from -249 to -17)	This study
pSG76 (2222-ΔRPE)	2222 construct with 99 nt deletion in in its 5' UTR (from -181 to -82)	This study

pSG77 (2222-ALL-RPE)	2222 construct with 68 nt insertion in its 5' UTR (from -247 to -181) containing RPE3 and part of RPE4 of uORF1´s 5' UTR (from -179 to -112)	This study
pSG78 (uORF2-ins68+4)	uORF2+4 construct with 68 nt insertion upstream of uORF4 (from -89 to -23) containing duplication of sequence upstream of *GCN4*´s start codon (from -90 to -24)	This study
pSG86 (uORF2-only-AAC)	low copy URA3 vector containing uORF2 only at its original position; G-129A, C-128A and G-109C substitutions were inserted upstream of uORF1	This study
pSG88 (uORF2-only-DELup39)	low copy URA3 vector containing uORF2 only at its original position; the sequences -181 to -143 upstream of uORF1 were deleted	This study
pSG89 (2222-SUB40)	2222 construct with -115 to -107 upstream sequences substituted by complementary sequences	This study
pSG90 (2222-CAAII)	2222 construct; the -143 to -122 upstream sequences were substituted by a stretch of CAA triplets of the identical length to the original sequence	This study
pSG91 (uORF2-only-SUB40)	low copy URA3 vector containing uORF2 only at its original position; the sequences -48 to -40 upstream of uORF1 were substituted by complementary sequences	This study
pSG93 (uORF2-only-CAAII)	low copy URA3 vector containing uORF2 only at its original position; the sequences -76 to -55 upstream of uORF1 were substituted by a stretch of CAA triplets of the identical length to the original sequence	This study
pSG100 (2222-ALL-RPE-AAC)	2222-ALL RPE construct; G-196A, C-195A and G-176C substitutions were inserted in its 5' UTR	This study
pSG102 (2222- ALL-RPE-DELup39)	2222-ALL RPE construct with 39 nt deletion in its 5' UTR (from -248 to -210)	This study
pSG138 (uORF2-only-SUB18)	low copy URA3 vector containing uORF2 only at its original position; the sequences -28 to -19 upstream of uORF2 were substituted by complementary sequences	This study

pSG141 (uORF2-only-CAAII-SUB18)	low copy URA3 vector containing uORF2 only at its original position; the mutations CAAII and SUB18 were combined	This study
pSG176 (uORF2-ins47CAA+4)	uORF2+4 construct with 47 nt insertion of CAA triplets upstream of uORF4 (from -69 to -23)	This study
pSG178 (uORF2-ins26CAA+4)	uORF2+4 construct with 26 nt insertion of CAA triplets upstream of uORF4 (from -33 to -23)	This study
pSG179 (uORF2+4-ins68)	uORF2+4 construct with 68 nt insertion upstream of *GCN4´*s start codon (from -178 to -112) containing duplication of sequence upstream of *GCN4´*s start codon (from -90 to -24)	This study
p180 (uORF1+2+3+4)	low copy URA3 vector containing wt *GCN4* leader	(15)
pSG237 (uORF1-Δ68+4)	uORF1+4 construct with 68 nt deletion downstream of uORF1 3´ sequences and upstream from uORF4 start codon (from -137 to -70)	This study
pSG244 (uORF1+2+4)	low copy URA3 vector containing uORF1, uORF2 and uORF4 only at their original positions	This study
YCp-a/TIF32-His-screen	single-copy a/TIF32-His with BamHI and NdeI sites introduced just in front of the start codon of a/TIF32 in LEU2 plasmid, from YCplac111	(12)
YCp-a/tif32-Box6+17-His	single-copy a/tif32-Box6+17- His in LEU2 plasmid, from YCplac111	(12)
YCp-a/tif32-Δ8-His	single-copy a/tif32-Δ8 -His in LEU2 plasmid, from YCplac111	(12)

Supplementary Table S3. Oligonucleotides used in this study.

Oligonucleotide	Sequence (5' to 3')
BS126	GGCTGATATTCGGACA
BS144	TCGGTCGACGGGGAATAAAG
SG9	GATTATTATTAGAAAATTATTAAGAGAATTATGTGTTAAATTTATTGAAAGAGAAAATTTATTTTC
SG10	GAAAATAAATTTTCTCTTTCAATAAATTTAACACATAATTCTCTTAATAATTTTCTAATAATAATC
SG11	GTTTTGTTACCAATTGCTATCATGTACCCGTAGAATTTTATTC
SG12	GAATAAAATTCTACGGGTACATGATAGCAATTGGTAACAAAAC
SG13	TGTTGTGAGTTTTTGTTTTGTTTTGTTTTG
SG14	CAAAACAAAACAAAACAAAAACTCACAACACACCAGCCACACAGCTCACTC
SG15	CGCAGATCTGGAAAATAAATTTTCTCTTTCAATAAATTTAACAC
SG16	CAAAACAAAACAAAACAAAAACTCACAACACTTGCTAAACCGATTATATTTTG
SG17	GGCAGATCTTTACGGAAACTTCTTGAATAAAATTCTACGG
SG18	GCAAGCTTTTTTCAATGATCTTTAATTTTTGTTGTGAGTTTTTGTTTTGTTTTG
SG19	AAAAATTAAAGATCATTGAAAAAAGCTTGC

SG20	CTTTCCGTAACGGTTACCTTTCTGTC
SG21	CCATCGATAATTTCACCGCCGAAAGGC
SG22	GACAGAAAGGTAACCGTTACGGAAAGATCTTG
SG25	ATTATTAAGAGAATTATGTGTTAAATTTATTGAAAGAG
SG26	CTCTTTCAATAAATTTAACACATAATTCTCTTAATAATTGTTGTGAGTTTTTGTTTTGTTTTG
SG27	AAAGATCATTGAAAAAAGCTTGCTAAAC
SG28	GTTTAGCAAGCTTTTTTCAATGATCTTTTGTTGTGAGTTTTTGTTTTGTTTTG
SG29	CGGTTTAGCAAGCTTTTTTCAATGATC
SG30	CTTGAGCAGACAAATTGGTAAACAAAACTTTAGTAATAATAATGATTTAATTAATGATAGTATAGGGACCTG ATAGCAATTGGTAACAAAAC
SG31	GTTTACCAATTTGTCTGCTCAAGTACCCGTAGAATTTTATTCAAGATG
SG47	GTTGTTGTTGTTGTTGTTGTTGTTGTTGTTGTTGTTGTTGTTGTTGTTGTTGTTGTTGTTGTTGTTGTCCTG ATAGCAATTGGTAACAAAAC
SG67	CAACAACAACAACAACAACAACAACTACCCGTAGAATTTTATTCAAGATG
SG85	CAATAAATTTAACACATAATTCTCTTAATAATTTTGATTATTATTTCTACTTTAAAAACAAAATATAATCGG
SG86	AAAATTATTAAGAGAATTATGTGTTAAATTTATTGAAAG
SG110	TAATTAAATCATTATTATTACTAAAGTTTTGTTTACCAATTTGTCTGCTCAAGTTTACTCGCCAATAAAAATT TCC

SG111	GGTAAACAAAACTTTAGTAATAATAATGATTTAATTAATGATAGTATAGGGACCTGGATAATTTGACAGAAAG
SG195	CACAGAATTCTCTTAATAATTTTCTAATAATAATC
SG196	GATTATTATTAGAAAATTATTAAGAGAATTCTGTGGTCAGTTTTTTGAAGAGTTATTTGTTTTG
VM7	ATCAGTTTCACTAGCGAATTATAC
VM8	GTATAATTCGCTAGTGAAACTGATTGTTGTGAGTTTTTGTTTTG
VM38	CAGTTTATTAAGTTATTATCAGTA
VM40	TACTGATAATAACTTAATAAACTGATTGTTGTTGTTGTTGTTGTTGTTGATTGCGAAGTAGATGAGTGAG
VM45	CCACACAGCTCACTCATCTACTTC
VM46	GAAGTAGATGAGTGAGCTGTGTGGGTGGTGAGTTGTATAATTCTTTAGTGAAACTGATGGGCAAAAAAA
VM72	ATTTTTTAATACGATACTGATAATTTGAATTATAACTGAACTAAAATAAAATATTTTG
VM73	ATTATCAGTATCGTATTAAAAAAT
